# Study on the efficiency of industry-university-research collaborative innovation in China based on a two-stage game cross-efficiency model incorporating Shapley value

**DOI:** 10.1371/journal.pone.0354999

**Published:** 2026-08-17

**Authors:** Xiaoqing Li, Xiaoling Ji

**Affiliations:** 1 School of Economics and Management, Xiamen University of Technology, Xiamen, China; 2 Scientific Research Department, Minnan Science and Technology College, Quanzhou, China; Gachon University, KOREA, REPUBLIC OF

## Abstract

This study evaluates the efficiency of China’s industry-university-research collaborative innovation (IURCI) system using a two-stage game cross-efficiency model integrated with Shapley values. By constructing an evaluation index system that captures the two stages of innovation: research and development (R&D) and technology transfer, and introducing the Shapley value to quantify the contributions of variables at each stage, this study addresses the limitations of traditional data envelopment analysis (DEA) models in weight allocation and competition handling. The empirical results indicate that: (1) China’s IURCI efficiency exhibits a clear pattern of high R&D efficiency but low transfer efficiency. (2) the central region demonstrates the highest overall efficiency with relatively balanced performance across both stages, while the eastern region leads in R&D but lags in technology transfer, and the western and northeastern regions experience pronounced stage imbalances. (3) innovation resource allocation tends to emphasize transfer while neglecting R&D with enterprises’ leading role in innovation insufficiently realized. Accordingly, this study recommends optimizing resource allocation mechanisms, strengthening the leadership role of enterprises, promoting the development of technology markets, and implementing differentiated regional policies to enhance the overall effectiveness of IURCI in China.

## 1 Introduction

Industry-university-research collaborative innovation (IURCI) refers to the systematic cooperation among enterprises, universities, and research institutes that integrates resources such as technology, talent, and capital to advance joint technological innovation, technology transfer and commercialization. The primary objective of IURCI is to eliminate barriers among innovation actors, foster knowledge sharing and complementary advantages, and accelerate the transformation of scientific and technological achievements into productive capacity. In contemporary China, IURCI has emerged as a vital engine driving industrial transformation and upgrading, playing a crucial role in supporting the nation’s transition toward high-quality economic development.

The efficiency of IURCI reflects a region’s capacity and potential to allocate innovation resources effectively and promote endogenous innovation growth. However, accurately assessing this efficiency remains challenging. Most current evaluations of the efficiency of industry-university-research collaboration overlook the varying contributions of innovation variables and the competitive relationships among innovation actors [1,2], making it difficult to accurately measure and analyze the efficiency of such collaboration. On the one hand, existing evaluations often overlook the unequal contributions of innovation variables and the competitive dynamics among innovation actors, which hinders precise measurement and analysis. Previous studies on IURCI efficiency frequently fail to fully account for the substantial differences in how various variables contribute to the collaborative innovation system [2]. This limitation arises mainly because IURCI is inherently a complex, multi-stage system involving multiple inputs and outputs. Conventional evaluation methods generally assume that all input variables contribute linearly and equally to outputs, without adequately examining the nonlinear and heterogeneous contribution mechanisms among variables. In practice, the relative contribution of each variable to the overall innovation efficiency can vary considerably across different stages of IURCI development [3,4]. On the other hand, most existing research does not adequately consider the direct and indirect competitive relationships faced by decision-making units (DMUs) in the process of IURCI development [5,6]. As China’s innovation-driven development strategy advances, provinces across the country have increased support for IURCI to strengthen regional innovation capabilities and promote industrial upgrading. This has intensified competition among provinces for innovation resources and collaborative opportunities among innovation entities. Such competitive dynamics profoundly affect the allocation of innovation resources, the formation of innovation partnerships, and the speed and quality of innovation outcomes [7,8].

This paper proposes a two-stage evaluation framework for IURCI that fully accounts for the heterogeneous contributions of different innovation variables within the system. It addresses the limitations of traditional evaluation approaches by incorporating the interaction effects among variables and the strategic behaviors of innovation actors. Specifically, a two-stage game cross-efficiency (GCE) model integrated with Shapley values is constructed. This model enables precise measurement of IURCI efficiency, provides an in-depth examination of the existing efficiency issues in China’s collaborative innovation system, and offers corresponding policy recommendations.The main contributions of this study are as follows: (1) It introduces the Shapley value from cooperative game theory into a two-stage DEA framework to objectively quantify the marginal contribution of each variable within the IURCI system, thereby enabling a more rational and equitable weighting of shared input and output indicators. (2) It develops an integrated model that combines Shapley value weighting with GCE model. This model simultaneously captures both the heterogeneity in variable contributions and the competitive interactions among DMUs, providing a more comprehensive representation of the co-opetition nature inherent in IURCI processes.

The remainder of this paper is structured as follows: Section 2 elaborates on innovation theory, the composition of indicator systems, and efficiency measurement methods related to IURCI. Section 3 presents the two-stage model of collaborative innovation and details the integration of the Shapley value method with the two-stage GCE model to construct the evaluation framework, along with the development of the indicator system and data sources. Section 4 analyzes the contributions of various variables within the IURCI system over the study period, examines the allocation ratio of shared input resources across the two stages, and evaluates the collaborative innovation efficiency of 30 Chinese provinces. Finally, Section 5 summarizes the research conclusions and provides policy implications.

## 2 Literature review

### 2.1 Innovation theory perspectives on IURCI

The essence of Industry-University-Research Collaborative Innovation (IURCI) lies in the systematic interaction between knowledge-producing and knowledge-applying actors. Evaluating its efficiency requires grounding in established innovation theories. This section reviews three interrelated streams of innovation theory—the Innovation Value Chain, Open Innovation and Absorptive Capacity, and the Triple Helix model—which collectively provide theoretical support for our two-stage modeling, variable contribution analysis, and regional efficiency comparisons.

Hansen and Birkinshaw (2007) [9] proposed the Innovation Value Chain (IVC) theory, decomposing innovation into three linked stages: idea generation, conversion, and diffusion. This decomposition directly justifies our two-stage structure, where the R&D stage corresponds to idea generation dominated by universities and research institutes, while the technology transfer stage corresponds to conversion dominated by firms. IVC theory emphasizes that resource allocation and coordination efficiency between stages determine overall innovation performance, underpinning our investigation of the “high R&D, low transformation” phenomenon. Subsequent research has extended IVC to regional innovation systems, showing that knowledge flow efficiency varies across regions and institutional contexts [10–12], reinforcing the need for stage-disaggregated measurement.

Complementing the structural perspective of IVC, the open innovation paradigm and absorptive capacity theory address the behavioral and organizational conditions for effective inter-organizational knowledge utilization. Chesbrough (2003) [13] defined open innovation as the systematic use of internal and external knowledge flows to accelerate innovation, with IURCI as a typical practice. Chesbrough et al. (2023) [14] argues that the greatest barriers to open innovation lie inside the organization, not outside. However, effective utilization of external knowledge depends on a firm’s absorptive capacity—the ability to recognize, assimilate, and apply external knowledge to commercial ends [15]. Cohen and Levinthal (1990) [15] demonstrated that absorptive capacity is largely a function of prior related knowledge and is path-dependent. Subsequent refinements have distinguished between potential and realized absorptive capacity and emphasized social integration mechanisms in knowledge transformation [16,17].

At the macro-institutional level, the Triple Helix model provides a complementary perspective on the broader environment shaping IURCI efficiency. Etzkowitz and Leydesdorff (2000) [18] proposed the Triple Helix model, emphasizing that university, industry, and government maintain distinct identities while overlapping functions co-evolve to drive innovation. In this model, innovation emerges from interactions among the three spheres, with each assuming roles traditionally associated with the others [19]. In regions with a mature Triple Helix structure, knowledge flows smoothly; in regions with helix misalignment, even strong R&D capacity may suffer from low transformation due to weak industrial absorption or policy incoordination. The Triple Helix thus provides a macro framework for our regional efficiency comparisons and differentiated policy recommendations.

Synthesizing the above perspectives, IVC offers a structural lens, open innovation and absorptive capacity address micro-level behavioral conditions, and the Triple Helix captures macro-institutional dynamics. Together, they provide a comprehensive theoretical foundation for understanding IURCI efficiency. However, they also leave two critical methodological questions unresolved: First, how can we objectively quantify each variable’s marginal contribution to the innovation system, accounting for the heterogeneous effects identified by absorptive capacity theory? Second, how can we model both cooperative and competitive relationships among decision-making units (DMUs) in efficiency evaluation, reflecting the co-evolutionary dynamics emphasized by the Triple Helix model?

### 2.2 Indicator systems and Shapley value applictions in IURCI efficiency evaluation

Most existing studies treat IURCI as an integrated whole, constructing indicator systems from input-output perspectives to evaluate collaborative innovation efficiency [20–22].With growing attention to the internal mechanisms, an increasing number of studies have begun to explore the internal structure of IURCI system, emphasizing that the efficiency assessment should fully consider the sequential relationships among subsystems by decomposing overall efficiency into multiple sub-stages [23,24]. Traditional Data Envelopment Analysis (DEA) black-box models fail to reveal the transformation processes of intermediate outputs or the allocation efficiency of input factors among subsystems, resulting in inconsistencies between measured and actual efficiency levels [25].

To overcome the limitations, two-stage DEA models and the more versatile network DEA models have been widely adopted. In banking, supply chain management, and manufacturing, these models have proven effective in capturing stage-specific efficiencies and inter-stage linkages [26–28]. Extending this approach to IURCI, researchers primarily decompose the R&D process into technology development and technology transfer stages [29–31]. This is consistent with the core characteristics of network DEA: multi-stage serialization and interdependence of intermediate variables. The two-stage framework developed in this paper constitutes a specific application of network DEA in the field of IURCI.

A key challenge is handling shared input variables (e.g., R&D personnel and funding) that affect both stages. Common allocation methods include equal proportion allocation [32] and principal component analysis [33]. The equal proportion approach assumes identical marginal contributions of input factors across stages, overlooking the nonlinear nature of innovation. PCA-based weighting fails to capture strategic interactions among innovation actors and neglects the synergistic 1 + 1 > 2 network effects inherent in collaborative innovation.

The Shapley value, proposed by Nobel laureate Lloyd Shapley (1952) [34], provides a theoretical framework for fair allocation in cooperative games. Its core principle lies in quantifying each participant’s marginal contribution across all possible coalitions. Shapley values are widely applied to assess variable contributions [35,36]. When used for weight determination, the Shapley value offers a robust method suitable for innovation networks characterized by strong synergy. Li and Liang (2010) [36] demonstrated that the Shapley value method is more robust and capable of capturing synergy effects. Abing et al. (2018) [37] employed a Shapley value-based multi-objective DEA to evaluate the academic efficiency of university departments. Wen (2020) [38] used Shapley values to calculate the marginal impact of variables on regional innovation efficiency, enabling rational selection of input–output indicators. Despite these advances, the application of Shapley values in IURCI efficiency studies remains limited, especially in multi-stage analyses involving shared input allocation. Thus, while existing research has acknowledged the importance of opening the black box and addressing shared inputs, the question of how to scientifically determine the allocation weights of these inputs across two stages remains a key unresolved issue.

### 2.3 Efficiency evaluation methods for IURCI

Current evaluation methods for IURCI efficiency primarily include parametric approaches (e.g., SFA) and non-parametric approaches (e.g., DEA). DEA, proposed by Charnes et al. (1978) [39], is widely adopted for its ability to handle multiple inputs and outputs without prespecifying a functional form. However, traditional DEA models assess efficiency through self-evaluation, allowing each DMU to choose weights that maximize its own efficiency. This leads to extreme weights and an inability to further rank efficient DMUs, resulting in superficial efficiency [5].

To address these shortcomings, various improvements have been proposed. Sexton et al. (1986) [40] introduced the cross-efficiency evaluation (CEE) model, which replaces self-evaluation with mutual evaluation among DMUs, mitigating extreme weighting and enabling ranking. Other methods include super-efficiency DEA [41] and inverse DEA [42,43], but both have limitations in multi-stage systems or are highly data-sensitive. This paper selects the CEE model because it incorporates mutual evaluation, aligns with the interactive relationships in IURCI, and facilitates integration with game theory.

However, Liang and Wu (2013) [5] noted that the CEE method suffers from non-unique optimal weights, yielding multiple solutions that are not Pareto optimal. Considering the direct and indirect competitive relationships among DMUs, Liang et al. (2008) [44] integrated game theory into the CEE framework, developing the game cross-efficiency (GCE) model. In this framework, DMUs are viewed as strategic players in a non-cooperative game, each seeking to enhance its own efficiency without diminishing that of others, converging to a Nash equilibrium.

In summary, although existing research has made significant progress in opening the black box of innovation, addressing shared inputs, and improving efficiency evaluation methods, no study has yet organically integrated the Shapley value for measuring variable marginal contributions with the GCE model for characterizing competitive interactions among DMUs to simultaneously address both issues. This is precisely the innovation of our study. Theoretically, the Shapley value provides a new perspective for allocating shared inputs in collaborative innovation. Methodologically, we construct a two-stage network DEA-GCE model based on Shapley value weighting. This model treats IURCI as a network system with internal structure, scientifically allocates shared inputs, and evaluates the competitive efficiency of each innovation entity within a non-cooperative game framework. It more accurately characterizes the complex co-opetition relationships in IURCI and offers a new methodological tool for efficiency evaluation in multi-stage complex systems.

## 3 Research methods

According to the innovation value chain theory [9], IURCI is a multi-stage process wherein innovation performance depends critically on the efficiency of both knowledge creation and knowledge conversion. To accurately capture this complexity, this study divides the IURCI process into two distinct stages: the research and development (R&D) stage and the technology transfer (TT) stage. However, as discussed in the literature review, two methodological challenges remain unresolved: how to quantify each variable’s marginal contribution to the innovation system, and how to model both cooperative and competitive relationships among decision-making units (DMUs) in efficiency evaluation.

To address these issues, this study employs the Shapley value and the DEA game cross-efficiency (GCE) model. The Shapley value, rooted in cooperative game theory, provides a rigorous mechanism for quantifying the nonlinear marginal contribution of each variable [34]. The GCE model, by treating DMUs as strategic players in a non-cooperative game that converges to a Nash equilibrium, captures the co-opetition dynamics among innovation actors [44]. Integrating these methods with the two-stage framework, a two-stage DEA-GCE model incorporating Shapley value weighting is constructed, which accounts for both the heterogeneous effects of innovation variables across different phases and the competitive interactions among DMUs.

### 3.1 Two-stage model of IURCI

**Fig 1:**
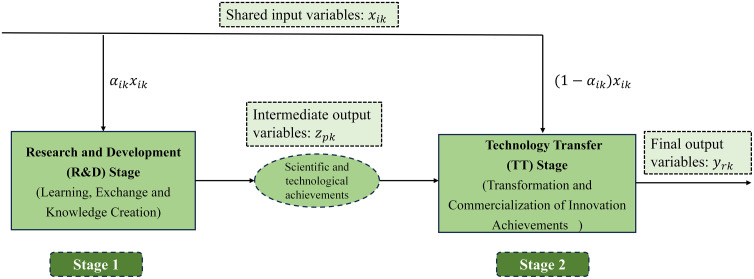
Two-stage system structure diagram of IURCI.

Based on the two fundamental stages of the innovation process, knowledge creation and value realization, this study subdivides IURCI activities into two distinct stages, namely the R&D stage and the TT stage (see Fig 1). The R&D stage is primarily led by universities and research institutes, which provide theoretical foundations, experimental facilities, frontier knowledge, and scientific personnel. This stage focuses on generating original breakthroughs in science and technology, addressing fundamental research questions and tackling key technological challenges. The TT stage, in contrast, is enterprise-driven and emphasizes the transformation of knowledge outputs from the R&D stage into scalable, market-oriented products or services, thereby realizing both economic and social value. During the R&D stage, the core innovation inputs, such as R&D funding and scientific personnel, are directed toward knowledge creation and technological advancement. Efficiency evaluation in this stage primarily emphasizes academic outputs (e.g., publications and patents), highlighting originality, feasibility, and scientific contribution. In the TT stage, however, investments such as pilot-scale testing funds, marketing expenses, and talent for industrialization are aimed at transforming the knowledge generated during the R&D stage into market-competitive products or services. Efficiency evaluations in this stage focus more on economic output and social benefits. Across the different stages of IURCI, there are significant differences in the roles of innovation inputs and outputs, as well as in how their efficiency is evaluated. Therefore, precise assessments must be achieved through the use of differentiated indicators and weighting.

In this two-stage system, the allocation of innovation resources is particularly critical. If resources are overly concentrated on R&D, it may delay the commercialization of results; if resources are allocated to commercialization too early, it may undermine the depth and originality of innovation. Therefore, it is essential to establish a mechanism that reflects the synergistic relationship between the two stages and enables the optimal allocation of resources. Drawing on the additive efficiency decomposition model [45], this study constructs a two-stage innovation system that incorporates shared inputs.

In this system (as shown in Fig 1), for $DMU_{k}(k=1,2,\cdots ,n)$, $x_{ik}(i=1,2,\cdots ,m)$ represents the shared input indicator for both stages, with allocation ratios of $\alpha_{ik}$ and $\left(1-\alpha_{ik}\right)$ in the first and second stages respectively. In addition to the shared input variable $(1-\alpha_{ik})x_{ik}$, the intermediate outputs $z_{pk}(p=1,2,\cdots ,q)$ from the first stage also serve as inputs for the second stage, ultimately forming s final outputs $y_{rk}\left(r=1,2,\cdots ,s\right)$. The proportion $\alpha_{ik}$ of the shared input indicator can be constrained by an upper bound $\alpha_{ik}^{U}$ and a lower bound $\alpha_{ik}^{L}$ based on actual settings. Drawing on the additive efficiency decomposition model [45], this paper decomposes the overall efficiency $E_{k}$ of the $DMU_{k}$ into a weighted average of the first-stage efficiency $E_{k}^{\text{1}}$ and the second-stage efficiency $E_{k}^{\text{2}}$, defined as follows:


$$\begin{array}{c} E_{k}=w_{k}^{\text{1}}E_{k}^{\text{1}}+w_{k}^{\text{2}}E_{k}^{\text{2}}=\frac{\sum_{p=1}^{q}w_{pk}z_{pk}+\sum_{r=1}^{s}u_{rk}y_{rk}}{\sum_{i=1}^{m}v_{ik}x_{ik}+\sum_{p=1}^{q}w_{pk}z_{pk}} \end{array}$$ (1)

$$\begin{array}{c} \begin{array}{c} E_{k}^{\text{1}}=\frac{\sum_{p=1}^{q}w_{pk}z_{pk}}{\sum_{i=1}^{m}v_{ik}\alpha_{ik}x_{ik}}\\ E_{k}^{\text{2}}=\frac{\sum_{r=1}^{s}u_{rk}y_{rk}}{\sum_{i=1}^{m}v_{ik}(1-\alpha_{ik})x_{ik}+\sum_{p=1}^{q}w_{pk}z_{pk}} \end{array} \end{array}$$ (2)

The two-stage weights $w_{k}^{\text{1}}$, $w_{k}^{\text{2}}$ represent the ratios of the inputs in each stage to the total inputs, i.e.,$w_{k}^{\text{1}}=\frac{\sum_{i=1}^{m}v_{ik}\alpha_{ik}x_{ik}}{\sum_{i=1}^{m}v_{ik}x_{ik}+\sum_{p=1}^{q}w_{pk}z_{pk}}$, $w_{k}^{\text{2}}=\frac{\sum_{i=1}^{m}v_{ik}(1-\alpha_{ik})x_{ik}+\sum_{p=1}^{q}w_{pk}z_{pk}}{\sum_{i=1}^{m}v_{ik}x_{ik}+\sum_{p=1}^{q}w_{pk}z_{pk}}$, and $w_{k}^{\text{1}}\text{+}w_{k}^{\text{2}}=1$. In the equation, $v_{ik}$, $w_{pk}$ and $u_{rk}$ denote theweights of the i-th shared input, the p-th intermediate output, and the r-th final output variable of $DMU_{k}$, respectively.

### 3.2 Shapley value

The Shapley value originates from cooperative game theory, with its core advantage being the objective quantification of the nonlinear contributions of different variables within collaborative systems based on the principle of marginal contribution. Drawing on some of the methods employed by Liu Yujia [35], this study calculates the Shapley values of each variable to measure their contribution to the efficiency of IURCI, and uses these values as the basis for weighting in subsequent models.

First, we define the Efficiency Change Ratio (ECR) to measure the marginal impact of a single variable or a combination of variables on efficiency. For a given $DMU_{k}$, consider its input indicator set I (containing all input variables). Let S be any non-empty subset of I (S⊆I,S≠∅), and let E(S) denote the optimal efficiency value obtained under the traditional DEA-CCR model for the input set S and the full output set R. Then, the ECR of the i-th input variable $x_{ik}$ is defined as:


$$\begin{array}{c} ECR\left(x_{i}\right)=\frac{E\left(S\cup \left\{x_{i}\right\}\right)-E\left(S\right)}{E\left(S\right)} \end{array}$$ (3)

Accordingly, the ECR of a variable subset T(T⊆I,T≠∅) is defined as the change in the efficiency of that subset relative to the efficiency when no inputs are used:


$$\begin{array}{c} ECR\left(T\right)=E\left(T\right)-E(\emptyset ) \end{array}$$ (4)

Here, E(∅) represents the efficiency when no inputs are provided, typically defined as 0 or a baseline value; for the sake of simplicity, we set it to 0 in this context. Based on this, the characteristic function V(T) of alliance T is defined as the sum of the efficiency changes of alliance T that do not include variable $x_{i}$:


$$\begin{array}{c} V\left(T\right)=\sum_{T\subseteq I\backslash \left\{x_{i}\right\}}ECR\left(T\right) \end{array}$$ (5)

The characteristic function V(T) satisfies superadditivity, meaning that the total payoff of the coalition is no less than the sum of the payoffs of its constituent subcoalitions, which lays the foundation for calculating the Shapley value. Consequently, the Shapley value contribution $\varphi_{i}$ of the input variable $x_{i}$


$$\begin{array}{c} \varphi_{i}=\sum_{T\subseteq I\backslash \left\{x_{i}\right\}}\frac{\left|T\right|!\left(\left|I\right|-\left|T\right|-1\right)!}{\left|I\right|!}\left[V\left(T\cup \left\{x_{i}\right\}\right)-V\left(T\right)\right] \end{array}$$ (6)

Here, |T| denotes the number of elements in alliance T, and |I| denotes the total number of elements in input set I. The Shapley value is unique; that is, for a given input set I and output set R, the contribution $\varphi_{i}$ of each variable $x_{i}$ is also uniquely determined. Finally, by normalizing the Shapley values of all variables, we obtain the weight coefficients $\lambda_{i}$ of each variable in the IURCI system:


$$\begin{array}{c} \lambda_{i}=\frac{\varphi_{i}}{\sum_{i\in I}\varphi_{i}} \end{array}$$ (7)

### 3.3 Two-stage GCE model incorporating Shapley value

The self-evaluation approach in traditional DEA models may lead to overestimation of efficiency values and prevent cross-comparisons. The GCE method introduces a peer-evaluation mechanism, requiring each DMU to use a unified weighting system when evaluating other units, thereby overcoming the shortcomings of self-evaluation in traditional DEA models. However, traditional GCE models do not account for the varying impacts of different variables on innovation systems. This study incorporates the Shapley value weights $\lambda_{i}$, calculated in Section 3.2, into the GCE model. These weights reflect the marginal contribution of variables within the collaborative innovation system, preventing the underestimation or overestimation of certain key variables and ensuring that the evaluation results are more persuasive and credible [38]. At the same time, the introduction of Shapley values embeds a cooperative logic into the competitive efficiency ranking, better aligning with the real-world scenario of coopetition and symbiosis in IURCI.

First, the Shapley values for each variable are normalized to obtain the weight coefficients $\lambda_{i}$ for each variable. These coefficients provide a clear measure of the actual impact of different variables—such as human resources and capital—on the innovation system. Next, $\lambda_{i}$ is incorporated into a two-stage GCE model. The model is constructed as follows:

Compared to $DMU_{d}$, the overall cross-efficiency $E_{dk}^{\left(g\right)}$ in the g-th round of the $DMU_{k}$ game can be obtained by solving the following optimization problem:


$$\begin{array}{c} \begin{array}{c} E_{dk}^{\left(g\right)}=\frac{\sum_{p=1}^{q}(w_{pk}^{d}{)}^{\left(g\right)}(\lambda_{pk}z_{pk})+\sum_{r=1}^{s}(u_{rk}^{d}{)}^{\left(g\right)}(\lambda_{rk}y_{rk})}{\sum_{i=1}^{m}(v_{ik}^{d}{)}^{\left(g\right)}(\lambda_{ik}x_{ik})+\sum_{p=1}^{q}(w_{pk}^{d}{)}^{\left(g\right)}(\lambda_{pk}z_{pk})}\\ s.t.\left\{ \begin{array}{c} @l@\frac{\sum_{p=1}^{q}(w_{pk}^{d}{)}^{\left(g\right)}(\lambda_{pk}z_{pk})}{\sum_{i=1}^{m}(v_{ik}^{d}{)}^{\left(g\right)}\alpha_{ik}(\lambda_{ik}x_{ik})}\leq 1;\\ \frac{\sum_{r=1}^{s}(u_{rk}^{d}{)}^{\left(g\right)}(\lambda_{rk}y_{rk})}{\sum_{i=1}^{m}(v_{ik}^{d}{)}^{\left(g\right)}\left(1-\alpha_{ik}\right)(\lambda_{ik}x_{ik})+\sum_{p=1}^{q}(w_{pk}^{d}{)}^{\left(g\right)}\left(\lambda_{pk}z_{pk}\right)}\leq 1;\\ \frac{\sum_{p=1}^{q}{\left(w_{pk}^{d}\right)}^{\left(g\right)}\left(\lambda_{pk}z_{pk}\right)+\sum_{r=1}^{s}{\left(u_{rk}^{d}\right)}^{\left(g\right)}\left(\lambda_{rk}y_{rk}\right)}{\sum_{i=1}^{m}{\left(v_{ik}^{d}\right)}^{\left(g\right)}\left(\lambda_{ik}x_{ik}\right)+\sum_{p=1}^{q}{\left(w_{pk}^{d}\right)}^{\left(g\right)}\left(\lambda_{pk}z_{pk}\right)}\geq E_{dk}^{\left(g-1\right)};\\ \alpha_{ik}^{L}\leq \alpha_{ik}\leq \alpha_{ik}^{U},i=1,2,\cdots ,m;k=1,2,\cdots ,n;\\ {\left(v_{ik}^{d}\right)}^{\left(g\right)},{\left(w_{pk}^{d}\right)}^{\left(g\right)},(u_{rk}^{d}{)}^{\left(g\right)}\geq 0,i=1,2,\cdots ,m;p=1,2,\cdots ,q;r=1,2,\cdots ,s. \end{array}\right. \end{array} \end{array}$$ (8)

In the above model, $(v_{ik}^{d}{)}^{\left(g\right)}$, $(w_{pk}^{d}{)}^{\left(g\right)}$ and $(u_{rk}^{d}{)}^{\left(g\right)}$ represent the weights for the i-th shared input variable, the p-th intermediate output variable, and the r-th final output variable of $DMU_{k}$ (relative to $DMU_{d}$) in the g-th game. $\lambda_{ik}$, $\lambda_{pk}$, $\lambda_{rk}$ denote the weight coefficients for the Shapley value contributions of the three variable types, shared inputs, intermediate outputs, and final outputs. $E_{dk}^{\left(g-1\right)}$ is the overall GCE efficiency of DMU in the (g−1)-th round of the game.

Model (8) is a nonlinear programming equation requiring two linear transformations. The first transformation involvesletting $\tau =1/\left[\sum_{i=1}^{m}(v_{ik}^{d}{)}^{\left(g\right)}(\lambda_{ik}x_{ik})+\sum_{p=1}^{q}(w_{pk}^{d}{)}^{\left(g\right)}\left(\lambda_{pk}z_{pk}\right)\right]$, then multiplying both the numerator and denominator of theobjective function by τ, and let $(\upsilon_{ik}^{d}{)}^{\left(g\right)}=\tau (v_{ik}^{d}{)}^{\left(g\right)}$, $(\omega_{pk}^{d}{)}^{\left(g\right)}=\tau (\omega_{ik}^{d}{)}^{\left(g\right)}$, $(\mu_{rk}^{d}{)}^{\left(g\right)}=\tau (u_{rk}^{d}{)}^{\left(g\right)}$. Next, perform a secondtransformation by setting $(\beta_{ik}^{d}{)}^{\left(g\right)}=(\upsilon_{ik}^{d}{)}^{\left(g\right)}\alpha_{ik}$, Which ultimately transforms model (8) into model (9).


$$\begin{array}{c} \begin{array}{c} maxE_{dk}^{\left(g-1\right)}=\sum_{p=1}^{q}(\omega_{pk}^{d}{)}^{\left(g\right)}(\lambda_{pk}z_{pk})+\sum_{r=1}^{s}(\mu_{rk}^{d}{)}^{\left(g\right)}(\lambda_{rk}y_{rk})\\ s.t.\sum_{i=1}^{m}(\upsilon_{ik}^{d}{)}^{\left(g\right)}(\lambda_{ik}x_{ik})+\sum_{p=1}^{q}(\omega_{pk}^{d}{)}^{\left(g\right)}(\lambda_{pk}z_{pk})=1;\\ \sum_{p=1}^{q}(\omega_{pk}^{d}{)}^{\left(g\right)}(\lambda_{pk}z_{pk})-\sum_{i=1}^{m}(\beta_{ik}^{d}{)}^{\left(g\right)}(\lambda_{ik}x_{ik})\leq 0;\\ \sum_{r=1}^{s}(\mu_{rk}^{d}{)}^{\left(g\right)}(\lambda_{rk}y_{rk})-\sum_{i=1}^{m}(\upsilon_{ik}^{d}{)}^{\left(g\right)}(\lambda_{ik}x_{ik})+\sum_{i=1}^{m}(\beta_{ik}^{d}{)}^{\left(g\right)}(\lambda_{ik}x_{ik})-\sum_{p=1}^{q}(\omega_{pk}^{d}{)}^{\left(g\right)}\left(\lambda_{pk}z_{pk}\right)\leq 0;\\ E_{dk}^{\left(g-1\right)}\cdot \sum_{i=1}^{m}{\left(\upsilon_{ik}^{d}\right)}^{\left(g\right)}\left(\lambda_{ik}x_{ik}\right)+(E_{dk}^{\left(g-1\right)}-1)\cdot \sum_{p=1}^{q}{\left(\omega_{pk}^{d}\right)}^{\left(g\right)}\left(\lambda_{pk}z_{pk}\right)-\sum_{r=1}^{s}{\left(\mu_{rk}^{d}\right)}^{\left(g\right)}\left(\lambda_{rk}y_{rk}\right)\leq 0;\\ \alpha_{ik}^{L}\leq \alpha_{ik}\leq \alpha_{ik}^{U},i=1,2,\cdots ,m;k=1,2,\cdots ,n;\\ {\left(\upsilon_{ik}^{d}\right)}^{\left(g\right)},{\left(\omega_{pk}^{d}\right)}^{\left(g\right)},(\mu_{rk}^{d}{)}^{\left(g\right)}\geq 0,i=1,2,\cdots ,m;p=1,2,\cdots ,q;r=1,2,\cdots ,s. \end{array} \end{array}$$ (9)

Using the above model (9), the overall cross-efficiency $E_{dk}^{\left(g\right)}$ of $DMU_{k}$ relative to $DMU_{d}$ in the g-th game, the weights $(\upsilon_{ik}^{d}{)}^{\left(g\right)}$, $(\omega_{pk}^{d}{)}^{\left(g\right)}$, $(\mu_{rk}^{d}{)}^{\left(g\right)}$, and the parameter $(\beta_{ik}^{d}{)}^{\left(g\right)}$ can be calculated.

The shared input allocation ratios $\alpha_{ik}$ and $\left(1-\alpha_{ik}\right)$ for shared inputs in the first and second stages can be calculated using model (10) and (11).


$$\begin{array}{c} \alpha_{ik}=\frac{(\beta_{ik}^{d}{)}^{\left(g\right)}}{(\upsilon_{ik}^{d}{)}^{\left(g\right)}} \end{array}$$ (10)

$$\begin{array}{c} 1-\alpha_{ik}=1-\frac{(\beta_{ik}^{d}{)}^{\left(g\right)}}{(\upsilon_{ik}^{d}{)}^{\left(g\right)}} \end{array}$$ (11)

Models (12) and (13) can be used to determine the efficiency values $E_{dk}^{1\left(g\right)}$, $E_{dk}^{2\left(g\right)}$ for the R&D stage and the TT stage, respectively, of $DMU_{k}$ relative to $DMU_{d}$ in the g-th game.


$$\begin{array}{c} E_{dk}^{1\left(g\right)}=\frac{\sum_{p=1}^{q}(\omega_{pk}^{d}{)}^{\left(g\right)}(\lambda_{pk}z_{pk})}{\sum_{i=1}^{m}{\left(\beta_{ik}^{d}\right)}^{\left(g\right)}\left(\lambda_{ik}x_{ik}\right)} \end{array}$$ (12)

$$\begin{array}{c} E_{dk}^{2\left(g\right)}=\frac{\sum_{r=1}^{s}(\mu_{rk}^{d}{)}^{\left(g\right)}(\lambda_{rk}y_{rk})}{\sum_{i=1}^{m}(\upsilon_{ik}^{d}{)}^{\left(g\right)}(\lambda_{ik}x_{ik})-\sum_{i=1}^{m}(\beta_{ik}^{d}{)}^{\left(g\right)}\left(\lambda_{ik}x_{ik}\right)+\sum_{p=1}^{q}{\left(\omega_{pk}^{d}\right)}^{\left(g\right)}\left(\lambda_{pk}z_{pk}\right)} \end{array}$$ (13)

Thus, we can obtain the overall cross-efficiency matrix $(E_{dk}^{0\left(g\right)}{)}_{n\times n}$ for $DMU_{k}$, the innovation R&D cross-efficiency matrix$(E_{dk}^{1\left(g\right)}{)}_{n\times n}$, and the technology transfer cross-efficiency matrix $(E_{dk}^{2\left(g\right)}{)}_{n\times n}$.

Furthermore, by taking the column averages of the cross-efficiency matrices, we obtain the overall efficiency $E_{k}^{\left(g\right)}$, innovation and R&D efficiency $E_{k}^{1\left(g\right)}$, and technology transfer efficiency $E_{k}^{2\left(g\right)}$ of $DMU_{k}$ in the g-th game:


$$\begin{array}{c} E_{k}^{\left(g\right)}=\frac{\text{1}}{n}\sum_{d=1}^{n}E_{dk}^{\left(g\right)},E_{k}^{1\left(g\right)}=\frac{\text{1}}{n}\sum_{d=1}^{n}E_{dk}^{1\left(g\right)},E_{k}^{2\left(g\right)}=\frac{\text{1}}{n}\sum_{d=1}^{n}E_{dk}^{2\left(g\right)} \end{array}$$ (14)

Repeat the above steps for all DMUs. When the overall cross-efficiency $E_{k}^{\left(g\right)}$, innovation R&D efficiency $E_{k}^{1\left(g\right)}$, and technology transfer efficiency $E_{k}^{2\left(g\right)}$ obtained by $DMU_{k}$ in the g-th game satisfy the difference between the overall efficiency $E_{k}^{\left(g-1\right)}$, innovation R&D efficiency $E_{k}^{1(g-1)}$, and technology transfer efficiency $E_{k}^{2(g-1)}$ obtained in the (g−1)-th game as defined by model (15), then all DMUs reach an equilibrium state.


$$\begin{array}{c} |E_{k}^{\left(g\right)}\text{-}E_{k}^{\left(g-1\right)}\text{|}<\varepsilon ,|E_{k}^{1\left(g\right)}-E_{k}^{1(g-1)}|<\varepsilon ,|E_{k}^{2\left(g\right)}-E_{k}^{2(g-1)}|<\varepsilon \end{array}$$ (15)

It is worth noting that the two-stage DEA game-theoretic cross-efficiency model incorporating Shapley values proposed in this paper derives its core algorithm from the game-theoretic cross-efficiency framework developed by Liang et al.(2008) [44], which has been proven to converge to a Nash equilibrium through iteration. The Shapley value weights introduced in the model are fixed coefficients calculated based on marginal contributions prior to iteration. They impose only linear constraints on the feasible region of the weights and do not affect the convexity and compactness of the original game model. Therefore, convergence remains theoretically guaranteed.

### 3.4 Index system and data sources

#### 3.4.1 Index system.

The efficiency of the IURCI indicator system constructed in this study is evaluated based on a set of input and output indicators (see Table 1). The input indicators represent shared investments across the R&D and TT stages, encompassing full-time equivalent R&D personnel as a measure of human capital input, internal R&D expenditure as a measure of financial input, and the number of industrial enterprises above a designated size engaged in R&D activities. The output indicators are divided into intermediate and final outputs. Intermediate outputs capture the achievements of the R&D stage, including the number of granted patents and published scientific papers. Final outputs reflect the economic outcomes of the TT stage, measured by sales revenue from new products and the volume of technology market transactions. The efficiency of these indicators is further analyzed using the Shapley value combined with the GCE model, which enables a more equitable and comprehensive evaluation of collaborative innovation performance.

**Table 1:** Evaluation index system for IURCI.

Stage	Variable	Indicators(Unit)	Symbol
R&D Stage	Shared input	Full-time equivalent of R&D personnel (Millennium Year)Internal expenditure of R&D funds (Ten thousand yuan)The number of large-scale industrial enterprises with R&D activities (Home)	$x_{\text{1}}$ $x_{\text{2}}$ $x_{\text{3}}$
R&D Stage /TT Stage	Intermediate output	The number of domestic patent authorizations (Item)The number of scientific and technological papers published (Article)	$z_{\text{1}}$ $z_{\text{2}}$
TT Stage	Final output	Transaction volume of the technology market (Ten thousand yuan) Sales revenue of new products (Ten thousand yuan)	$y_{\text{1}}$ $y_{\text{2}}$

#### 3.4.2 Data sources.

The data used in this study are primarily obtained from the China Statistical Yearbook (2014–2023) and the China Science and Technology Statistical Yearbook (2014–2023). The research sample covers 30 provinces in China, with the study period spanning from 2015 to 2022. Considering the inherent time lag between inputs and outputs in IURCI activities, this study adopts a two-year lag period for both input and output indicators, consistent with the methodology employed in previous studies [32].

## 4 Results

### 4.1 IURCI variables’ contribution

This study employs Matlab R2021a to calculate the Shapley value (contribution levels) of seven variables within the IURCI system for the years 2015–2022. These values are normalized to derive corresponding weight coefficients for each variable (see Table 2). The magnitude and fluctuations in variable contributions reflect their differentiated roles across different stages, providing more refined decision-making support for optimizing innovation resource allocation.

**Table 2:** IURCI variables’ contribution.

Year / Variable		$x_{1}$	$x_{2}$	$x_{3}$	$z_{1}$	$z_{2}$	$y_{1}$	$y_{2}$
2015	Shapley	4.6404	3.8105	0.5039	2.1501	9.5200	0.7976	17.1169
	Sum	38.5394						
	Weight coefficient	0.1204	0.0989	0.0131	0.0558	0.2470	0.0207	0.4441
2016	Shapley	5.7165	4.2909	0.6867	2.4501	10.7570	1.3354	1.1230
	Sum	26.3596						
	Weight coefficient	0.2169	0.1628	0.0261	0.0929	0.4081	0.0507	0.0426
2017	Shapley	7.3236	6.5971	0.9842	1.2948	15.0048	3.0794	0.6744
	Sum	34.9583						
	Weight coefficient	0.2095	0.1887	0.0282	0.0370	0.4292	0.0881	0.0193
2018	Shapley	8.5895	8.0500	2.0604	1.4170	10.9703	3.5204	0.5650
	Sum	35.1726						
	Weight coefficient	0.2442	0.2289	0.0586	0.0403	0.3119	0.1001	0.0161
2019	Shapley	9.4928	7.8096	11.1340	1.0857	6.1873	4.3754	0.6067
	Sum	40.6915						
	Weight coefficient	0.2333	0.1919	0.2736	0.0267	0.1521	0.1075	0.0149
2020	Shapley	10.1369	7.2514	0.7503	1.4286	6.1602	7.2177	0.1660
	Sum	33.1111						
	Weight coefficient	0.3061	0.2190	0.0227	0.0431	0.1860	0.2180	0.0050
2021	Shapley	10.4726	6.3950	0.2923	1.8583	6.1434	5.4977	0.1438
	Sum	30.8031						
	Weight coefficient	0.3400	0.2076	0.0095	0.0603	0.1994	0.1785	0.0047
2022	Shapley	8.9530	7.7121	0.4458	2.7939	5.9392	3.3973	0.1490
	Sum	29.3903						
	Weight coefficient	0.3046	0.2624	0.0152	0.0951	0.2021	0.1156	0.0051

From the perspective of innovation input variables, the weight coefficients for full-time equivalent R&D personnel ($x_{\text{1}}$) and internal R&D expenditure ($x_{\text{2}}$) exhibited an overall upward trend, emerging as dominant factors particularly during the period from 2020 to 2022. This finding indicates that the sustained accumulation of human resources and financial investment is essential for the long-term enhancement of IURCI efficiency. In contrast, the contribution of industrial enterprises above a designated size engaged in R&D activities ($x_{\text{3}}$) experienced a sharp increase only in 2019 (weight coefficient 0.2736), likely resulting from the temporary strengthening of enterprises’ leading role in innovation under policy incentives. Nevertheless, the role of enterprises as the primary innovation actors within the IURCI system remains relatively weak. With respect to intermediate innovation inputs, the marginal contribution of scientific and technological paper publications ($z_{\text{2}}$) surpassed that of patent authorizations ($z_{\text{1}}$). The weight coefficient for paper publications ($z_{\text{2}}$) reached its peak at 0.4292 in 2017 and subsequently declined to 0.2021 in 2022, suggesting a gradual weakening of the direct contribution of academic publications to the innovation system. Regarding innovation output variables, the contribution of technology market transaction volume ($y_{\text{1}}$) has shown a steady upward trend. As technology market mechanisms continue to mature, market dynamics have progressively become the main driver of efficiency improvement. Overall, the enhancement of China’s IURCI efficiency currently depends primarily on R&D human resources and financial investment, academic paper output, and the vitality of technology transaction markets. However, the driving force contributed by enterprises remains unstable and underutilized, while universities and research institutions continue to serve as the central actors in R&D activities. From the perspective of absorptive capacity theory [15–17], the low and volatile contribution of the enterprise-related variable suggests that many firms lack sufficient in-house R&D to effectively recognize, assimilate, and commercialize knowledge generated by universities and research institutes. This provides a theoretical explanation for our finding that the dominant role of enterprises as innovation actors has not been fully realized.

### 4.2 Allocation ratios of shared input resources at two stages

By employing the two-stage GCE model incorporating Shapley value and undergoing multiple game iterations, the average allocation ratios of the three shared input variables during the R&D stage and the TT stage can be calculated. The results are presented in Table 3.

**Table 3:** Proportions of shared input resources distributed in two stages.

Region	Province	R&D Stage	TT Stage
Eastern region	Beijing	46.21%	53.79%
	Tianjin	41.42%	58.58%
	Hebei	39.24%	60.76%
	Shanghai	39.43%	60.57%
	Jiangsu	38.52%	61.48%
	Zhejiang	45.07%	54.93%
	Fujian	43.30%	56.70%
	Shandong	36.08%	63.92%
	Guangdong	55.16%	44.84%
	Hainan	57.59%	42.41%
	Mean value	44.20%	55.80%
Central region	Shanxi	37.42%	62.58%
	Anhui	40.13%	59.87%
	Jiangxi	43.52%	56.48%
	Henan	39.89%	60.11%
	Hubei	36.42%	63.58%
	Hunan	35.72%	64.28%
	Mean value	38.85%	61.15%
Western region	Inner Mongolia	36.44%	63.56%
	Guangxi	48.69%	51.31%
	Chongqing	41.93%	58.07%
	Sichuan	48.61%	51.39%
	Guizhou	56.87%	43.13%
	Yunnan	41.18%	58.82%
	Shaanxi	42.66%	57.34%
	Gansu	48.84%	51.16%
	Qinghai	46.54%	53.46%
	Ningxia	44.41%	55.59%
	Xinjiang	60.76%	39.24%
	Mean value	46.99%	53.01%
Northeast Region	Liaoning	36.58%	63.42%
	Jilin	54.09%	45.91%
	Heilongjiang	54.77%	45.23%
	Mean value	48.48%	51.52%
National average value		44.58%	55.42%

Innovation resource allocation across China’s 30 provinces generally exhibits a pattern of prioritizing technology transfer (TT) over research and development (R&D) with investments in the TT stage (55.42%) substantially exceeding those in the R&D stage (44.58%). According to the innovation value chain (IVC) theory [9–12], an unbalanced allocation of resources between knowledge creation (R&D stage) and knowledge conversion (transformation stage) can lead to overall innovation inefficiency. The observed pattern indicates a systemic bias toward short-term commercialization at the potential expense of long-term foundational research capacity.

Regionally, the imbalance between the two stages is more pronounced in eastern and central China than in the western and northeastern regions. In particular, central China allocates, on average, more than 20% additional resources to commercialization than to R&D, possibly reflecting its urgency to catch up with the eastern economies through accelerated market application. The significant imbalance observed in eastern regions may stem from their well-established industrial bases, where enterprises prioritize the rapid commercialization of R&D outputs. In contrast, the smaller gap in the western and northeastern provinces likely arises from incomplete innovation chains or limited foundational R&D capacities, which constrain large-scale investment differences between the two stages. Overall, China’s innovation resource allocation demonstrates a distinctly market-oriented tendency. However, the regional disparities identified through the Shapley value combined with the GCE model highlight the necessity of region-specific policy adjustments to balance short-term commercialization efficiency with the long-term development of R&D capabilities

### 4.3 Efficiency of IURCI

This study employs the two-Stage GCE model incorporating Shapley value. The empirical calculation adopts a convergence criterion that the absolute difference of overall and sub-stage efficiency between two adjacent iterations is less than 10^−4^. All DMUs can achieve convergence, and the obtained results are stable and reliable. On this basis, this study accurately measure the overall IURCI efficiency as well as the efficiency values of R&D and TT stages in 30 Chinese provinces and municipalities from 2015 to 2022 (see Table 4).

**Table 4:** Average efficiency of China’s IURCI from 2015 to 2022.

Region	Province	Overall efficiency	Efficiency of R&D Stage	Efficiency of TT Stage
Eastern region	Beijing	0.8691	0.9336	0.8454
	Tianjin	0.7600	0.8716	0.7149
	Hebei	0.8013	0.9177	0.7573
	Shanghai	0.8349	0.9197	0.8050
	Jiangsu	0.7698	0.9284	0.7041
	Zhejiang	0.8187	0.9160	0.7765
	Fujian	0.6259	0.9072	0.4825
	Shandong	0.7387	0.7846	0.7228
	Guangdong	0.8448	0.8772	0.8346
	Hainan	0.6140	0.9855	0.3507
	Mean value	0.7677	0.9042	0.6994
Central region	Shanxi	0.7417	0.9041	0.6798
	Anhui	0.9008	0.9551	0.8801
	Jiangxi	0.9352	0.9709	0.9225
	Henan	0.7216	0.9596	0.6252
	Hubei	0.9069	0.9567	0.8910
	Hunan	0.8533	0.8433	0.8626
	Mean value	0.8433	0.9316	0.8102
Western region	Inner Mongolia	0.6590	0.8822	0.5739
	Guangxi	0.8750	0.9853	0.8168
	Chongqing	0.8684	0.9409	0.8383
	Sichuan	0.6537	0.9702	0.4914
	Guizhou	0.6924	0.9946	0.4942
	Yunnan	0.5441	0.9468	0.3435
	Shaanxi	0.7893	0.9744	0.7037
	Gansu	0.7037	0.9821	0.5458
	Qinghai	0.8062	0.9882	0.7074
	Ningxia	0.6761	0.9636	0.5318
	Xinjiang	0.6857	0.9871	0.4774
	Mean value	0.7231	0.9650	0.5931
Northeast Region	Liaoning	0.7380	0.9020	0.6753
	Jilin	0.9373	0.9747	0.9185
	Heilongjiang	0.6326	0.9884	0.3948
	Mean value	0.7693	0.9550	0.6629
National average value		0.7666	0.9371	0.6789

Analysis of IURCI efficiency across China’s 30 provinces from 2015 to 2022 reveals significant regional disparities and developmental imbalances in overall performance. The national average efficiency is 0.7666, with R&D stage efficiency (0.9371) markedly higher than that of the technology transfer (TT) stage (0.6789). This can be understood through the lens of the innovation value chain and absorptive capacity. IVC theory posits that strong performance in knowledge creation does not automatically translate into effective knowledge conversion, rather the latter requires complementary absorptive capacity and institutional support [9–11,15]. The substantial gap between R&D efficiency and transformation efficiency suggests a bottleneck at the interface between the two stages. For an extended period, China’s science and technology policy framework and research evaluation mechanisms have prioritized quantifiable indicators such as patent authorizations and scientific publications. This emphasis has inherently biased innovation resource allocation toward the R&D stage, inflating intermediate outputs and artificially elevating R&D efficiency. However, the successful commercialization of scientific achievements depends on mature venture capital systems, robust intellectual property protection, strong corporate technology absorption capacity, and well-functioning interest-linkage mechanisms among universities, industries, and research institutes. Deficiencies in these areas reveal structural weaknesses in China’s innovation ecosystem, leading to insufficient final outputs and low conversion efficiency. The empirical results derived from the Shapley value combined with the GCE model further substantiate this conclusion. As discussed in Section 4.1, the declining contribution of academic papers alongside the rising weight of technology market transactions reflects a policy shift from emphasizing academic output volume to prioritizing market value. Nonetheless, the inertia of the entrenched institutional system continues to constrain overall transformation efficiency.

Regionally, the central region exhibits the highest overall and stage-specific efficiencies, both surpassing the national average, demonstrating the greatest two-stage equilibrium. The superior and balanced performance of central China aligns well with the Triple Helix model [18]. A mature Triple Helix structure-characterized by functional overlap and synergistic co-evolution among universities, industry, and government—facilitates smooth knowledge flows and coordinated resource allocation. The “*Rise of Central China*” strategy may have fostered such a balanced triple-helix configuration. In contrast, despite its agglomeration of top-tier universities and R&D resources, eastern China exhibits a transformation efficiency (0.6994) substantially lower than its R&D efficiency (0.9042). This finding is consistent with concepts such as “knowledge filter” and “knowledge flow” in the extended literature on open innovation [46–48], where barriers such as weak intellectual property protection, mismatched incentive structures, or cultural differences between academia and industry impede the flow of knowledge from creators to users.Western and northeastern China also exhibit a pattern of high R&D efficiency but low transformation efficiency, with an even larger gap between the two stages.This can be explained by absorptive capacity theory [15–17]. These regions host nationally prominent research universities and institutes (e.g., Xi’an, Chengdu, Harbin), yet their local industrial bases lack sufficient internal R&D capacity to absorb and commercialize the abundant academic knowledge. This creates innovation islands where knowledge is produced locally but commercialized elsewhere, reflecting a structural misalignment between knowledge creation and industrial absorption. Overall, China’s IURCI development must transition from a R&D-heavy approach to a collaboration-driven model. Policy guidance should aim to optimize resource allocation across both stages, particularly by addressing conversion inefficiencies in the central, western, and northeastern regions to foster balanced coordination between innovation and industrial chains.

Globally, comparative analysis underscores this need for transformation. The United States emphasizes robust market mechanisms and venture capital systems; Germany’s Fraunhofer model serves as a benchmark for IUR collaboration by focusing on applied research tailored to industrial application; and Japan’s innovation system is predominantly enterprise-led. In contrast, China’s IURCI model remains largely policy-driven, with the government playing a central role in resource allocation and strategic guidance. While this approach has accelerated R&D capacity development in the short term, further refinement of market mechanisms remains essential. International experience indicates that R&D investment alone does not automatically ensure efficient commercialization. Only through optimized resource allocation, strengthened market mechanisms, enhanced enterprise leadership in innovation, improved science and technology services, and equitable benefit-sharing mechanisms can China achieve a deep integration of its innovation and industrial chains—advancing from an innovation powerhouse to an innovation superpower.

## 5 Conclusions and policy suggestions

### 5.1 Conclusions

This study employs the improved two-stage GCE model combined with the Shapley value to systematically evaluate the IURCI efficiency of China’s 30 provinces from 2015 to 2022, while also examining the contributions of various innovation system variables and regional disparities. The main findings are as follows:

(1) China’s collaborative innovation efficiency demonstrates a distinct pattern of high R&D and low technology transfer. Efficiency in the R&D stage is significantly higher than that in the technology transfer stage, suggesting strong resource utilization capabilities in knowledge creation and technological development but substantial deficiencies in transforming scientific achievements into marketable and industrialized outcomes.
(2) the contributions of innovation input and output variables to overall efficiency exhibit significant heterogeneity and temporal dynamics. The contribution of R&D investment has shown a continuous upward trend, becoming a major driver of efficiency improvement. However, enterprises, as the core actors in innovation, have yet to fully leverage their potential, displaying only intermittent strengthening effects under policy incentives. Meanwhile, the contribution of scientific paper output has gradually declined, whereas the influence of technology market transaction volume has steadily increased—reflecting a structural shift in China’s innovation system from an academically oriented to a market-driven model.
(3) the allocation of innovation resources reveals a general tendency to prioritize technology transfer over R&D, with the proportion of national resources devoted to the TT stage exceeding that of the R&D stage. Regional heterogeneity in resource allocation strategies is evident: the central region allocates the highest share of resources to transformation activities, followed by the eastern and western regions, while the northeastern region maintains a relatively balanced distribution. This pattern reflects both differences in regional innovation development stages and divergent policy orientations, while also exposing structural inconsistencies between foundational R&D capabilities and transformation demands in certain regions.
(4) regional efficiency performance varies markedly across provinces. The central region exhibits the highest overall efficiency, characterized by relatively balanced performance across both stages. The eastern region leads in R&D efficiency but lags behind in TT efficiency, constraining its overall performance. In contrast, the western and northeastern regions display pronounced two-stage imbalances, typified by high R&D but low technology transfer.

### 5.2 Policy suggestions

Based on the above conclusions derived from the Shapley value combined with the GCE model, this paper proposes the following policy recommendations to enhance the efficiency of IURCI in China:

(1) Optimize innovation resource allocation mechanisms. Innovation value chain (IVC) theory advocates for flexible resource allocation mechanisms that dynamically balance investment between the R&D and transformation stages based on regional development stages. The government should move beyond the entrenched tendency to prioritize technology transfer over R&D. By establishing more flexible resource allocation and profit-sharing mechanisms, policymakers can achieve a better balance between short-term economic returns and long-term innovation capacity. In particular, western and northeastern regions should moderately increase investment in the R&D stage to reinforce their foundational innovation capacity. Meanwhile, targeted incentives—such as the creation of dedicated technology transfer funds and preferential tax policies—should be introduced to encourage enterprises to expand investment in pilot testing and industrialization.
(2) Strengthen the role of enterprises as the core drivers of innovation. Absorptive capacity theory suggests that policies should prioritize strengthening firms’ in-house R&D capabilities, especially in western and northeastern regions, through R&D subsidies and tax incentives. Specifically, Enterprises should be encouraged to lead the formation of innovation consortia, participate deeply in university and research institute R&D activities, and align innovation objectives with market demand. Mechanisms such as open innovation challenges and targeted R&D commissions can help enterprises articulate technological needs, engage in project design and outcome evaluation, and enhance synergy throughout the innovation chain.
(3) Improve the technology market and commercialization service system. Efforts should be made to accelerate the development of a unified, open, standardized, and efficient technology transaction market to improve transparency in scientific achievements and strengthen supply–demand matching. The role of third-party institutions should be expanded to provide professional services such as intellectual property valuation, technology brokerage, and pilot-scale refinement, thereby reducing transaction costs and uncertainties associated with commercialization.
(4) Implement regionally differentiated innovation policies. The Triple Helix model implies that governments should act as facilitators rather than directors, promoting cross-sectoral platforms where universities, industry, and government co-design innovation agendas. The eastern region should focus on dismantling institutional barriers between R&D and commercialization to foster deep integration of innovation and industrial chains. The central region can capitalize on its balanced development advantages to establish demonstration zones for IURCI. The western and northeastern regions should prioritize capacity building by improving technology conversion platforms, cultivating local technology service markets, and narrowing commercialization gaps.
(5) Establish long-term collaboration and benefit-sharing mechanisms. A Shapley value–based revenue distribution framework should be adopted to rationally define and quantify the contributions of universities, research institutions, and enterprises across different stages of the innovation process. By implementing equitable intellectual property sharing and profit-distribution arrangements, stakeholders’ rights and incentives can be safeguarded, thereby enhancing the sustainability and stability of collaborative innovation.

### 5.3 Limitations

There may be discrepancies between model assumptions and real-world scenarios. Although this study partially addresses issues of variable contribution disparities and agent competition through Shapley value and GCE model, it does not fully account for the impact of external factors such as environmental changes, policy interventions, and international technological cooperation. Future research could incorporate methods like three-stage network DEA and synthetic control approaches to control for external environments and random disturbances, thereby enhancing the accuracy of efficiency measurement.

Although this study introduces methodological innovations, it does not provide a direct numerical comparison with traditional two-stage DEA models using the same dataset. Future research could conduct systematic comparisons and quantitative improvements to validate the model’s effectiveness and superiority. Additionally, the impact of different assumptions regarding the allocation of shared inputs on efficiency measurement results could be explored, thereby providing a more robust empirical basis for evaluating the efficiency of collaborative innovation among industry, academia, and research institutions.

## Supporting information

S1 Data Data set 2015–2022data. (XLSX)
