# Supplementary material for: Study on the efficiency of industry-university-research collaborative innovation in China based on a two-stage game cross-efficiency model incorporating Shapley value
Source: PLoS One. 2026 Aug 17;21(8):e0354999. doi: 10.1371/journal.pone.0354999 (PMC13480662; doi:10.1371/journal.pone.0354999)
Supplement: Supplementary file 1 [file pone.0354999.s002.docx]

Study on the Efficiency of Industry-University-Research Collaborative Innovation in China Based on An Improved Two-Stage Game Cross-Efficiency Model Incorporating Shapley Value

The manuscript is very interesting, the topic is relevant and the innovation of the manuscript comes from the uses of Shapley values on Two-Stage GCE model.

In terms of methodological innovation, the manuscript incorporates the concept of the Shapley value in IURCI and constructs an improved GCE model integrated with this Shapley value. In methodological terms the manuscript explains in detail all the steps. Indeed, the results are accurate.

The main recommendation is the discussion of the results that are missing and the authors should examine the results and compare them with the other literature results.

Please note along the text in various pages the authors forgot to insert the (year) after the cited reference. Please kindly see: Page 11 line 89 Li Yongjun et al. Please insert (year)

The same after Bingzhou.... Charnes et al…..Sexton et al……on the next pages and all over along the manuscript

Best wishes!
