# Supplementary material for: Study on the efficiency of industry-university-research collaborative innovation in China based on a two-stage game cross-efficiency model incorporating Shapley value
Source: PLoS One. 2026 Aug 17;21(8):e0354999. doi: 10.1371/journal.pone.0354999 (PMC13480662; doi:10.1371/journal.pone.0354999)
Supplement: Supplementary file 2 [file pone.0354999.s004.docx]

Respone to Reviewers

**I would like to express my sincere gratitude to the reviewers for their feedback on this manuscript. Below, I will address the comments from the three reviewers one by one.**

**Reviewer #1**：

1. The Introduction is generally well written and clearly motivates the study, but it relies too heavily on single citations to support several authoritative statements. In most cases, key conceptual and contextual claims should be supported by more than one relevant reference to demonstrate adequate engagement with the literature and to strengthen the credibility of the arguments. While multiple citations are not required for every statement, foundational claims would benefit from broader citation support.

Response: We appreciate the reviewer’s comments. In response to your feedback, we have reorganized the introduction section. While retaining the original research motivation, core logical framework, and research focus, we have added five high-quality relevant references (specifically, references [1, 2, 4, 6, 7] in this paper) to the existing literature [3, 5, 8]. These additions are primarily intended to further support the foundational, conceptual, and contextual arguments presented in the paper. The supplementary evidence provided by these new references has further enriched the breadth and depth of our literature review, fully demonstrating our in-depth examination of existing research findings. This has effectively enhanced the credibility and strength of our arguments, thereby effectively addressing the previous limitation of the introduction’s narrow range of citations.

2. There are formatting inconsistencies throughout the manuscript that require careful correction. In particular, inconsistent spacing appears after full stops, as observed in the first paragraph of Section 2.1. In addition, many citations lack a space between the final word and the opening square bracket, and citation lists within brackets do not consistently include spaces after commas, for example “[10,11]” instead of “[10, 11]”. These issues should be revised consistently across the entire manuscript to meet standard academic formatting conventions.

Response: To address the formatting issues in this section, we have conducted a thorough, section-by-section and sentence-by-sentence review of the entire manuscript and made consistent revisions: First, we focused on correcting inconsistencies in punctuation and spacing throughout the text to ensure uniformity in punctuation usage and spacing; Second, we conducted a comprehensive review of all citations, adding missing spaces between the final word of a quotation and the opening square bracket. We also standardized the formatting of the citation list within square brackets and corrected issues with the reference citation format to ensure compliance with standard academic norms; Third, we simultaneously reviewed the entire text for any other potential inconsistencies in formatting, implementing comprehensive corrections to effectively enhance the document’s formal rigor.

3. The first paragraph in Section 2.2 is excessively long and should be divided to improve readability and clarity. More generally, the authors are advised to ensure that no paragraph exceeds approximately 12 lines throughout the manuscript, as overly long paragraphs hinder comprehension and weaken the presentation.

Response: In response to your feedback, we have optimized and refined the content of Section 2.2: First, we have reasonably restructured the originally lengthy first paragraph of Section 2.2 to ensure that the paragraph length aligns with your recommendations, significantly improving the readability and clarity of the content; Second, building on the paragraph optimization, we have supplemented the research methods and developmental context of the hyper-efficiency model and the inverse DEA model, further enriching the theoretical foundation of the research methods section and making the argumentation more systematic and in-depth (see lines 136-147 of the manuscript); Third, in the final paragraph of Section 2.2, we have reorganized and refined the innovative aspects of this study, clearly highlighting its core contributions to enable reviewers and readers to quickly grasp the key research highlights (see lines 157-168 of the manuscript).

4. Two-stage and network DEA models have been extensively applied across a wide range of domains, particularly in banking, supply chain management, and other service and production systems. The literature review would benefit from a paparagraph or more dedicated to discussion of these broader applications and how such models have been used to capture internal structures and inter-stage relationships before narrowing the focus to industry–university–research collaborative innovation. This would help situate the proposed approach within the wider DEA application landscape and clarify its relevance and transferability to the present study.

Response: In response to your suggestion, we have added a second paragraph to Section 2.1 (see lines 77-94 of the manuscript) specifically to elaborate on the widespread applications and core roles of two-stage and network DEA models: We clearly articulate the core value of these two models in overcoming the “black box” limitations of traditional DEA and capturing the internal structure of the system as well as the interrelationships among various stages. We discuss their specific applications in the banking sector (measuring the efficiency of various operational stages and aiding operational optimization), supply chain management (characterizing coordination mechanisms among supply chain nodes and optimizing resource allocation), and other service and production systems (such as healthcare and manufacturing, where they identify efficiency bottlenecks). We have cited seven relevant references [17-23] to support these points; At the same time, the text naturally transitions to the field of industry-academia-research collaborative innovation, explaining how the core strengths of these models align with the two-stage R&D process of such collaboration (technology development and technology transfer). It clarifies that the two-stage efficiency measurement framework constructed in this paper represents a concrete application of these models in this field.

5. In line 108, the reference to Sexton et al. is not accompanied by a corresponding bracketed citation. Please add the appropriate square-bracket citation to ensure the source can be clearly and consistently referenced.

Response: The correction has been made on line 134 of the manuscript.

6. A similar issue occurs in line 112, where Liang is mentioned without a corresponding bracketed citation. In addition, the same author is inconsistently referred to as “Liang” and “Liang Liang” across lines 110 and 112. These inconsistencies indicate insufficient attention to reference accuracy and citation consistency, and they should be carefully corrected throughout the manuscript.

Response: We have implemented targeted corrections regarding this issue: First, we have standardized the citation format for the two relevant papers by Professor Liang Liang, supplementing and refining them in strict accordance with academic citation standards. The specific revisions can be found on lines 148 and 150 of the manuscript; We have standardized the author’s name, correcting the inconsistent references to “Liang” and “Liang Liang” throughout the text to “Liang Liang” to ensure consistency in author attribution; second, taking this as an opportunity, we have conducted a comprehensive review and standardized revision of the citations and author attribution throughout the entire manuscript, focusing on issues such as missing citations, inconsistent author names, and non-compliant citation formats, to prevent such problems from recurring.

7. The manuscript focuses on cross-efficiency as a means of distinguishing among efficient DMUs but does not acknowledge or briefly discuss alternative approaches commonly used for this purpose, such as super-efficiency, inverse DEA, or others. While it is acceptable to concentrate on a single method, the authors should at least provide a concise discussion of other established ranking techniques and clarify which improvement they are making.

Response: In response to your suggestion, we have added relevant content to the second paragraph of Section 2.2 (lines 136-147), specifically to discuss alternative methods for ranking efficient DMUs and to justify the rationale behind the method selected in this paper: We clearly introduce two commonly used methods for efficient DMU ranking—the super-efficiency DEA model and the inverse DEA model—and explain the core principles, advantages, and limitations of each: the super-efficiency model is simple to compute and highly interpretable, but struggles to account for the interdependent effects across different stages of a multi-stage system; the inverse DEA model provides specific optimization directions but is highly sensitive to data and prone to being influenced by outliers; At the same time, the paper clearly explains the rationale for selecting the cross-efficiency model, emphasizing its advantage of incorporating a mutual evaluation mechanism among DMUs. This approach not only aligns with the interactive relationships among various entities in industry-academia-research collaborative innovation but also facilitates integration with game theory. It addresses the shortcomings of the super-efficiency and inverse DEA models in depicting entity interactions, thereby clearly highlighting the rationality of the methodological choice and the value of the proposed improvements, making the justification of the research methodology more comprehensive and persuasive.

8. In lines 121–123, the manuscript refers to “subjectivity inherent in traditional weighting methods,” but this claim is not adequately explained or justified. The authors should clarify in what sense these weighting approaches are subjective, for example by explaining whether subjectivity arises from analyst-imposed weights, arbitrary assumptions, or unrestricted weight flexibility in DEA. Without such clarification, the statement remains vague and should either be properly substantiated or revised.

Response: Since the “subjectivity inherent in traditional weighting methods” mentioned in the manuscript is not the primary distinction between the new model and traditional models, we have revised the analysis in the literature review section to highlight key points and strike an appropriate balance between detail and brevity, thereby clarifying the description of the paper’s contributions and innovations.

9. Figure 1 is visually weak and does not meet the presentation quality expected for a journal article. The figure should be enlarged, with increased font sizes to ensure readability. If possible, the authors are encouraged to use color and clearer visual differentiation between components to enhance clarity and overall visual appeal.

Response: In response to your suggestion, we have revised Figure 1. The revised version can be found on line 196 of the manuscript: First, we have enlarged the image to ensure greater overall clarity. Second, we have increased the font size within the image to address the issue of poor readability, making it easier for reviewers and readers to clearly view the content. Third, in accordance with the journal’s requirements, we have optimized the visual presentation of the figure, enhanced the visual distinction between its various components, and further improved its clarity and overall appearance to ensure it meets the image requirements of《PLOS ONE》.

10. The proposed framework clearly follows a network DEA structure, with multiple stages connected through intermediate outputs, yet the manuscript does not explicitly use the term “network DEA” to characterize the model. The authors should clarify this choice and explain whether the omission is intentional. If the model indeed aligns with the network DEA paradigm, this should be stated explicitly to avoid conceptual ambiguity and to position the contribution more accurately within the DEA literature.

Response: It should be clarified that the two-stage efficiency evaluation framework constructed in this paper is essentially an extension of the network DEA model. By decomposing the collaborative innovation process among industry, academia, and research institutions into two sequential stages—technology R&D and technology transfer—it accurately captures the internal flow mechanisms of the innovation process. The previous lack of explicit use of the term “network DEA” may have led to ambiguity regarding the model’s theoretical positioning. In this revised version, we clarify its connection to network DEA, further highlighting the research method’s positioning and value within the DEA literature.

11. Between lines 157–184, the description of the proposed model is insufficiently explicit and should be expanded to better explain the model structure, underlying assumptions, and feasibility. In addition, typographical issues are present, particularly in line 181, where unusual symbols appear between terms instead of standard commas. These errors should be corrected. The authors should also provide clear and complete definitions of all variables and parameters used, and explicitly explain how the equations are logically connected to improve clarity and reproducibility. In addition, some equation numbers are missing. Please revise all, especially how the network is connected and its feasibility.

Response: In response to the issues you raised, we have completed a systematic review and revision: First, we have thoroughly reviewed and optimized this section, removing redundant content, streamlining the wording, and expanding the model description; second, we have corrected formatting issues by replacing non-standard symbols used between terms with standard symbols, and simultaneously identified and corrected other potential formatting inconsistencies in this paragraph; Third, we have re-edited the model content, revising and refining all variables and parameters used in the model, and clearly explaining the logical relationships between the equations to make the model’s derivation more coherent and its structure clearer; Fourth, we have added missing equation numbers to ensure consistent and standardized numbering across all equations, further enhancing the rigor of the manuscript.

12. For many of the equations, including Equations (5), (6), (7), and others, the definitions of terms are incomplete or unclear. All symbols, indices, and exponents must be explicitly defined. For instance, in Equation (7), the expression includes s, yet only is defined, with no explanation provided for the exponent . Such omissions undermine the clarity and rigor of the mathematical formulation and should be corrected consistently throughout the manuscript.

Response: In response to your comments, we have completed a systematic revision of the research model section in Chapter 3: We have focused on reorganizing and optimizing the model and related content in Sections 3.1, 3.2, 3.3 (see lines 170–274 of the manuscript), while also conducting a comprehensive review of all term definitions and variable symbols throughout the manuscript. We have supplemented and refined each one to ensure that all symbols, indices, and exponents in the equations are clearly and explicitly explained, thereby thoroughly correcting omissions such as the unexplained exponent in Equation (7). Furthermore, we have conducted a comprehensive review of the mathematical expressions throughout the manuscript to ensure consistency and standardization in the definitions of terms and explanations of variable symbols. This has effectively enhanced the clarity and academic rigor of the mathematical presentation and prevents such issues from recurring.

13. The manuscript engages a broad DEA methodological space, yet the reference base appears relatively limited for that scope (approximately 24 citations). The literature review and methodological positioning would be strengthened by incorporating more recent and authoritative DEA sources, including key works on network/two-stage DEA, shared-input allocation, cross-efficiency, and game cross-efficiency, as well as recent applications in innovation efficiency contexts. I encourage the authors to expand the bibliography with high-quality journal articles and foundational methodological papers to better demonstrate coverage of the state of the art and to justify the selected modelling choices. You may consider reading and citing the DEA related papers below as they relate to the subject matter.

Inverse DEA for Portfolio Volatility Targeting: Industry Evidence from Taiwan Stock Exchange. International Journal of Financial Studies, 13(4), 192. https://doi.org/10.3390/ijfs13040192

An Integrated Approach to Stock Selection and Ranking: Combining Shannon Entropy Technique, DEA, and Inverse DEA [Article]. Ieee Access, 13, 138851-138866. https://doi.org/10.1109/ACCESS.2025.3586417

Explainable DEA–ensemble approach with golden jackal optimization: efficiency evaluation and prediction for United States information technology firms. Machine Learning with Applications, 23, 100798. https://doi.org/https://doi.org/10.1016/j.mlwa.2025.100798

Response: We have carefully reviewed and screened the DEA-related literature you suggested and recommended. The first two papers are more closely aligned with the research content of the inverse DEA section of this paper and effectively supplement the theoretical and practical arguments regarding inverse DEA. Therefore, we have included these two papers in the references of this paper to strengthen the supporting framework of the inverse DEA section.

**Reviewer #2:**

1.The main recommendation is the discussion of the results that are missing and the authors should examine the results and compare them with the other literature results.

Response: In response to your suggestion, we have revised and expanded the Results and Discussion section. The specific changes are as follows: On lines 365–367 of the manuscript, we have incorporated the relevant research paper by Wang Shouwen et al. (2025) and conducted a detailed comparative analysis of our study’s findings with the conclusions of that literature. This comparison serves to further validate the authenticity and reliability of our findings—the core results of this study, including the efficiency characteristics of IURCI and the patterns of regional differentiation, effectively align with the conclusions of Wang Shouwen et al., thereby substantiating the scientific rigor of our methodology and the validity of our findings.

2. Please note along the text in various pages the authors forgot to insert the (year) after the cited reference. Please kindly see: Page 11 line 89 Li Yongjun et al. Please insert (year)
3. The same after Bingzhou.... Charnes et al…..Sexton et al……on the next pages and all over along the manuscript

Response: In response to this issue, we have systematically reviewed and thoroughly checked the citation format throughout the entire manuscript. We focused on examining all the citations you mentioned, as well as other references in the text. We have revised the citation format for the relevant references by adding the publication year after the author’s name, ensuring that the year is consistently included for all authors. This ensures a uniform and standardized citation format and resolves the issue of missing publication years in the references throughout the manuscript.

**Reviewer #3:**

1. Some references are missing that would benefit the paper:

Yongjun Li, Liang Liang,, A Shapley value index on the importance of variables in DEA models, Expert Systems with Applications, Volume 37, Issue 9, 2010, Pages 6287-6292, ISSN 0957-4174, https://doi.org/10.1016/j.eswa.2010.02.093.

Qingxian An, Yao Wen, Tao Ding, Yongli Li, Resource sharing and payoff allocation in a three-stage system: Integrating network DEA with the Shapley value method, Omega,

Volume 85, 2019, Pages 16-25, ISSN 0305-0483,

https://doi.org/10.1016/j.omega.2018.05.008.

Fanyong Meng, Li Wu, Junfei Chu, Allocating the fixed cost based on data envelopment analysis in view of the Shapley value, Expert systems, Volume37, Issue5 Special Issue:Advances in visual analytics and mining visual data, 2020, e12539, https://doi.org/10.1111/exsy.12539

Abing, S.L.N., Barton, M.G.L., Dumdum, M.G.M. et al. Shapley value-based multi-objective data envelopment analysis application for assessing academic efficiency of university departments. J Ind Eng Int 14, 733–746 (2018). https://doi.org/10.1007/s40092-018-0258-6

Response: Regarding the screening and inclusion of references, the details are as follows: This paper focuses on the core issue of “determining the allocation of indicator weights” in the evaluation of the efficiency of industry-academia-research collaborative innovation using Shapley values. Among the recommended references, the first and fourth papers are highly relevant to the research topic of this paper and can effectively supplement and support its research content. The second and third papers primarily focus on the application of the Shapley value in benefit allocation and cost allocation. Considering the relevance of the research content in the references and the limited scope of this paper, only the first and fourth papers have been included in the reference list.

2.Section 2.2 Where is exactly the novelty with respect to other contributions in the literature stated at “Building on these insights, the present study introduces two major innovations. Theoretically, it incorporates the concept of the Shapley value from cooperative game theory into innovation efficiency evaluation. ”

Response: In response to your inquiry, we have revised the introduction to clarify the core novelty of this paper relative to existing literature and its specific innovative contributions. Please refer to lines 54 through 59 for details.

3.3.3 Improved Two-Stage GCE Model Incorporating Shapley Value: is the convergence of the model guaranteed?

Response: We sincerely appreciate your highly insightful and valuable feedback. We take your concerns regarding the model’s convergence very seriously and fully recognize the importance of supplementing and refining this aspect to enhance the paper’s rigor and highlight its research value. We have provided additional clarification in the final paragraph of Section 3.3 of the paper; please refer specifically to the explanation of the convergence of the Shapley-value-based two-stage DEA game cross-efficiency model. (See lines 269–274 of the manuscript.) Specifically, the convergence of the model constructed in this paper is fully guaranteed from three perspectives: theoretical foundation, model design, and empirical validation:

First, the core algorithm of the model is derived from the game-theoretic cross-efficiency framework proposed by Liang et al. (2008). This classical framework has been rigorously proven through theoretical derivations; it converges to a Nash equilibrium through iterative calculations based on mutual evaluation games among decision units, providing a solid theoretical foundation for the convergence of our model. (See lines 269–271 of the manuscript.)

Second, the Shapley value weights introduced in this paper do not participate in the iterative game process. Instead, they are fixed coefficients calculated based on the marginal contributions of each variable prior to iteration. They impose only linear constraints on the feasible region of the weights, without altering the convexity and compactness of the original game model. Therefore, they do not affect the model’s inherent convergence properties, ensuring the theoretical continuity of convergence; (See lines 271–274 of the manuscript)

Third, during the empirical calculations, we explicitly defined the termination condition for the iteration—the maximum difference between the overall efficiencies of two consecutive iterations must be less than 10⁻⁴. Through repeated verification, all decision-making units were found to converge within a finite number of steps (with an average of approximately 25 iterations). The empirical results further validate the stability and reliability of the model’s convergence. (See lines 265–268 of the manuscript)

4. Results As stated previously, comparison with previous two stage models must be done

Response: Thank you very much for your valuable feedback. Regarding the comparative analysis with the unmodified model (the traditional two-stage DEA model), we fully agree with your perspective; such a comparison is crucial for validating the effectiveness of the model improvements. The focus of this study is on proposing a new model that integrates Shapley value with GCE model and applying it to the evaluation of collaborative innovation efficiency among IUR in China. Due to space constraints, we have not yet conducted parallel calculations using the traditional two-stage model; however, we have indirectly highlighted the advantages of our improved model through various means:

On the one hand, in the literature review section, we systematically identified the core limitations of the traditional two-stage DEA model, including extreme weight allocation, neglect of competitive interactions among decision units, and subjective allocation of shared inputs. This theoretically established the direction and necessity of our model’s improvements; On the other hand, a comparison with the conclusions of studies using traditional DEA models indirectly confirms the superiority of our model—traditional DEA models often result in multiple DMUs having an efficiency value of 1, making it impossible to further distinguish between efficient DMUs.

For example, a study by Qiu Dongfang and Hu Zhengping [1] (based on the traditional DEA model) showed that among China’s 30 provinces from 2007 to 2011, eight provinces had an efficiency value of 1 for IUR collaboration, making it impossible to distinguish differences in their efficiency; In the study by Zhou Guangliang et al. [2], the traditional two-stage DEA model indicated that 19 provinces had a comprehensive efficiency of 1 in the stage of scientific and technological output, and 16 provinces had a comprehensive efficiency of 1 in the stage of technology transfer; Yang Yulu’s [3] study compared the traditional DEA-BCC model with the traditional two-stage DEA model with shared inputs, and both models showed multiple decision units with an efficiency of 1. In contrast, the new model constructed in this paper effectively overcomes this limitation, accurately capturing the core characteristic of “high R&D, low commercialization” and the regional differentiation patterns in IURCI. These key findings are often smoothed over or obscured in traditional model studies, fully demonstrating the value of this model’s improvements. To further enhance the rigor of the study, we will add a new discussion section in the conclusion of the revised manuscript (see lines 442–447 of the manuscript), explicitly noting that future research could conduct parallel comparative studies of various models to quantify the extent of this model’s improvements and address the shortcomings of existing research.

**References**:

[1]Qiu Dongfang, Hu Zhengping. Efficiency and Sustainability of Industry-University-Research Collaboration at the Provincial Level in China: Based on Provincial Panel Data and the DEA-Malmquist Productivity Index Method [J]. Technology and Economy, 2013, 32(12): 82-89.

[2] Zhou Guangliang, Zhao Congyu, Chen Yu, et al. Performance Evaluation and Spatial Pattern Analysis of Industry-University-Research Collaborative Innovation in China: Based on Two-Stage DEA and the Malmquist Index [J]. Journal of Xinyang Normal University (Natural Science Edition), 2022, 35(02): 217-223.

[3] Yang Yulu. Evaluation of Industry-University-Research Efficiency in Chinese Provinces, Municipalities, and Autonomous Regions Based on Two-Stage Shared-Input DEA [J]. Journal of Wenzhou University (Natural Science Edition), 2023, 44(02): 27-37.

5.Minor details , Please, check for minor typos. Even the title has some spelling mistakes.

Response: Thank you for your feedback. We have reviewed the entire text for spelling errors and made corrections based on your comments.
