# Supplementary material for: Study on the efficiency of industry-university-research collaborative innovation in China based on a two-stage game cross-efficiency model incorporating Shapley value
Source: PLoS One. 2026 Aug 17;21(8):e0354999. doi: 10.1371/journal.pone.0354999 (PMC13480662; doi:10.1371/journal.pone.0354999)
Supplement: Supplementary file 3 [file pone.0354999.s005.docx]

**Response to Reviewers**

**1. Do you really believe that a sufficient discussion and comparison was done with just a single additional reference? In result analysis section, the references supporting the authors’ arguments is generally insufficient. Conduct the discussion and comparison by including a variety of existing studies. This applies to entire Section 4.**

**Response：**We sincerely accept your comments and have revised and expanded Chapter 4 accordingly. The revision no longer relies on a limited number of references for discussion; instead, we have incorporated a broad range of existing relevant studies and conducted comprehensive comparative analysis and in-depth discussion.

In the revised chapter, we systematically explain the imbalance in two-stage resource allocation and the heterogeneous characteristics of regional efficiency by integrating innovation value chain theory, triple helix theory, open innovation theory, and absorptive capacity theory. Meanwhile, we introduce cutting-edge research findings on knowledge barriers, knowledge flow, and regional innovation development, and conduct horizontal comparisons with the empirical results of this study. We have supplemented sufficient literature support for the research conclusions and improved the comparative discussions with existing studies throughout Chapter 2, further enhancing the academic rigor and persuasiveness of the result analysis.

**2. Do you really believe that you have corrected the typo in the title?**

**Response：**Yes, we have carefully corrected the spelling error in the title. In addition, we have conducted a comprehensive spelling check and proofreading throughout the entire manuscript to ensure that no similar errors appear.

**3.Avoid excessive use of quotation marks. It gives the impression that the manuscript is a product of GenAI. I do not believe that any of the currently used quotation marks are necessary.**

**Response：**We fully agree with this comment. We have removed all unnecessary quotation marks throughout the manuscript and standardized the usage of all punctuation marks in strict accordance with academic writing conventions**.**

**4.Although this journal is at the SCIE level, most of the references cited are either not SCIE-level or are limited to domestic journals in China. Except for the critical research, please replace most of the references with SCIE-level ones. As a current manuscript, concerns about the reliability and quality of the references are raised.**

**Response：**Thank you very much for your valuable comments. We have comprehensively optimized the reference list, replaced and removed some domestic Chinese journal articles, and supplemented high-quality SCIE-level literature. The revised 42 references include 12 Chinese articles and 30 English articles, which we hope will enhance the academic quality and reliability of the citations.

**5.Although this research is regarding IURCI, there is no literature review on innovation theory to support the research. Please add innovation studies literature as a subsection of Literature, and also use them in the result analysis and discussion parts.**

**Response：**Thank you very much for your pertinent suggestions. We have added a new Subsection 2.1 titled “Innovation theory perspectives on IURCI ”in the literature review chapter. This subsection systematically supplements three core theories that are highly consistent with IURCI: innovation value chain theory, open innovation and absorptive capacity theory, and triple helix theory. Meanwhile, the relevant theoretical content has been reasonably integrated into the results and discussion section, so as to consolidate the theoretical foundation of this study.

6. **I do not believe that the name of Sections 2.1 and 2.2 are appropriate. Please revise them to accurately represent the main text.**

**Response：**We have carefully adopted this revision suggestion. The title of the original Section 2.1 (now renumbered as Section 2.2) has been revised to Indicator systems and Shapley value applictions in IURCI efficiency evaluation. The original Section 2.2 (now renumbered as Section 2.3) has also been revised and renamed as“ **Efficiency evaluation methods for IURCI”**.

**5.As there are many mathematical formulas, please review once more to ensure that formulas including superscripts and subscripts have been written correctly.**

**Response：**We have thoroughly checked and organized all mathematical formulas throughout the manuscript. The subscripts, superscripts, and various symbol formats have been verified one by one to ensure that all formulas are written in a standardized and accurately expressed manner.

**6.In Table 2, the variability of the weighting coefficients across years is quite large. In order to ensure the reliability of these results, please consider additional tests.**

**Response：**Thank you very much for your valuable comments. The large differences in the Shapley value weights across years in Table 2 are objective results calculated from the actual data. We have rechecked and recalculated the raw data and confirmed that these differences are not caused by measurement errors. In addition, we have explained the underlying reasons for such differences in the manuscript. The fluctuations in the weights stem from real changes in various innovation input and output indicators. The substantial differences in certain years are mainly due to drastic short-term changes in some indicators driven by policy adjustments, which led to sharp increases in their corresponding weights. For example, as mentioned in Session 4.1: “In contrast, the contribution of industrial enterprises above a designated size engaged in R&D activities （$x_{3}$）experienced a sharp increase only in 2019 (weight coefficient 0.2736), likely resulting from the temporary strengthening of enterprises’leading role in innovation under policy incentives”. Through repeated calculations and analyses, we confirm that the weight coefficients presented in Table 2 are reasonable and reliable.

**7.Indicate the number of game iterations.**

**Response：**In the empirical calculation process, a clear convergence criterion is established in advance: when the absolute differences of both the overall efficiency and the sub-stage efficiencies between two consecutive iterations are all less than 10^-4^, all DMUs are considered to have reached a convergent state, and the calculation results are stable and reliable. It should be noted that the actual number of iterations for each DMU is not exactly the same, so a unified number of iterations cannot be provided. Once the above convergence condition is met, the iteration process terminates automatically.
