# Supplementary material for: Study on the efficiency of industry-university-research collaborative innovation in China based on a two-stage game cross-efficiency model incorporating Shapley value
Source: PLoS One. 2026 Aug 17;21(8):e0354999. doi: 10.1371/journal.pone.0354999 (PMC13480662; doi:10.1371/journal.pone.0354999)
Supplement: Supplementary file 4 [file pone.0354999.s006.docx]

**Response to Reviewers**

1. If the reviewer comments include a recommendation to cite specific previously published works, please review and evaluate these publications to determine whether they are relevant and should be cited. There is no requirement to cite these works unless the editor has indicated otherwise.

**Response：**Thank you for your reminder. We have thoroughly combed through the entire manuscript to ensure that only references highly relevant to this research are cited.

2. Please review your reference list to ensure that it is complete and correct. If you have cited papers that have been retracted, please include the rationale for doing so in the manuscript text, or remove these references and replace them with relevant current references. Any changes to the reference list should be mentioned in the rebuttal letter that accompanies your revised manuscript. If you need to cite a retracted article, indicate the article’s retracted status in the References list and also include a citation and full reference for the retraction notice.

**Response：**Thank you for your valuable comments. We have thoroughly double-checked all references throughout the manuscript and confirmed that no retracted literature has been cited.

3.All issues are relevantly addressed, except for literature reviews on innovation. Only limited papers were merely mentioned for each innovation theory, which was not expected when I left comment. While it would be beneficial to enrich the relevant discussions in each subsection, rather I propose merging all sub-sections into section 2.1 only and then literature on innovation should be logically reviewed in a single section. Final acceptance will be on the condition that the aforementioned issue is sufficiently addressed.

**Response：**Thank you for your valuable comments. We have reorganized the content of Section 2.1 Innovation theory perspectives on IURCI in the original manuscript. The innovation theories distributed across the three subsections are integrated into a unified section, and additional key supporting literatures are supplemented accordingly. The revised passages can be found on Lines 70-106, Line166-178,Line 319, Line 330, Line 356 and Line 380 of the revised manuscript.

**Data Availability**

The panel dataset supporting the findings of this study covers indicators of industry-university-research collaborative innovation across 2015-2022, with all raw data sourced from China Statistical Yearbook. The dataset has been publicly deposited in Figshare , with the unique Figshare identifier **https://doi.org/10.6084/m9.figshare.32955860**.

This dataset contains all indicator variables for IURCI from 2015 to 2022, as well as executable codes for replicating the two-stage DEA game cross-efficiency and Shapley value model calculations.

The ORCID ID information of the authors is listed as follows:

**Xiaoling Ji: ORCID ID: 0009-0008-0124-0870;**

**Xiaoqing Li: ORCID ID: 0009-0008-3095-010X.**
