# Supplementary material for: Study on the efficiency of industry-university-research collaborative innovation in China based on a two-stage game cross-efficiency model incorporating Shapley value
Source: PLoS One. 2026 Aug 17;21(8):e0354999. doi: 10.1371/journal.pone.0354999 (PMC13480662; doi:10.1371/journal.pone.0354999)
Supplement: Peer review history

# Peer review: Decision Letter 0

**DOI:** 10.1371/journal.pone.0354999.r001

**Authors:** Hongbum Kim

2 Feb 2026

Dear Dr. Ji,

Thank you for submitting your manuscript to PLOS ONE. After careful consideration, we feel that it has merit but does not fully meet PLOS ONE’s publication criteria as it currently stands. Therefore, we invite you to submit a revised version of the manuscript that addresses the points raised during the review process.

Please submit your revised manuscript by Mar 19 2026 11:59PM. If you will need more time than this to complete your revisions, please reply to this message or contact the journal office at plosone@plos.org. When you're ready to submit your revision, log on to https://www.editorialmanager.com/pone/ and select the 'Submissions Needing Revision' folder to locate your manuscript file.

- A letter that responds to each point raised by the academic editor and reviewer(s). You should upload this letter as a separate file labeled 'Response to Reviewers'.
- A marked-up copy of your manuscript that highlights changes made to the original version. You should upload this as a separate file labeled 'Revised Manuscript with Track Changes'.
- An unmarked version of your revised paper without tracked changes. You should upload this as a separate file labeled 'Manuscript'.

If you would like to make changes to your financial disclosure, please include your updated statement in your cover letter. Guidelines for resubmitting your figure files are available below the reviewer comments at the end of this letter.

If applicable, we recommend that you deposit your laboratory protocols in protocols.io to enhance the reproducibility of your results. Protocols.io assigns your protocol its own identifier (DOI) so that it can be cited independently in the future. For instructions see: https://journals.plos.org/plosone/s/submission-guidelines#loc-laboratory-protocols. Additionally, PLOS ONE offers an option for publishing peer-reviewed Lab Protocol articles, which describe protocols hosted on protocols.io. Read more information on sharing protocols at https://plos.org/protocols?utm_medium=editorial-email&utm_source=authorletters&utm_campaign=protocols.

We look forward to receiving your revised manuscript.

Kind regards,

Hongbum Kim, Ph.D.

Academic Editor

PLOS One

Journal Requirements:

When submitting your revision, we need you to address these additional requirements.

1. Please ensure that your manuscript meets PLOS ONE's style requirements, including those for file naming. The PLOS ONE style templates can be found at

https://journals.plos.org/plosone/s/file?id=wjVg/PLOSOne_formatting_sample_main_body.pdf and

https://journals.plos.org/plosone/s/file?id=ba62/PLOSOne_formatting_sample_title_authors_affiliations.pdf

2. Please note that PLOS One has specific guidelines on code sharing for submissions in which author-generated code underpins the findings in the manuscript. In these cases, we expect all author-generated code to be made available without restrictions upon publication of the work. Please review our guidelines at https://journals.plos.org/plosone/s/materials-and-software-sharing#loc-sharing-code and ensure that your code is shared in a way that follows best practice and facilitates reproducibility and reuse.

3. When completing the data availability statement of the submission form, you indicated that you will make your data available on acceptance. We strongly recommend all authors decide on a data sharing plan before acceptance, as the process can be lengthy and hold up publication timelines. Please note that, though access restrictions are acceptable now, your entire data will need to be made freely accessible if your manuscript is accepted for publication. This policy applies to all data except where public deposition would breach compliance with the protocol approved by your research ethics board. If you are unable to adhere to our open data policy, please kindly revise your statement to explain your reasoning and we will seek the editor's input on an exemption. Please be assured that, once you have provided your new statement, the assessment of your exemption will not hold up the peer review process.

4. Please upload a copy of Figure 3-1, to which you refer in your text on page 8. If the figure is no longer to be included as part of the submission please remove all reference to it within the text.

5. If the reviewer comments include a recommendation to cite specific previously published works, please review and evaluate these publications to determine whether they are relevant and should be cited. There is no requirement to cite these works unless the editor has indicated otherwise.

[Note: HTML markup is below. Please do not edit.]

Reviewers' comments:

Reviewer's Responses to Questions

**Comments to the Author**

1. Is the manuscript technically sound, and do the data support the conclusions?

Reviewer #1: Yes

Reviewer #2: Yes

Reviewer #3: No

**********

2. Has the statistical analysis been performed appropriately and rigorously?

Reviewer #1: Yes

Reviewer #2: Yes

Reviewer #3: No

**********

3. Have the authors made all data underlying the findings in their manuscript fully available?

The PLOS Data policy

Reviewer #1: Yes

Reviewer #2: Yes

Reviewer #3: Yes

**********

4. Is the manuscript presented in an intelligible fashion and written in standard English?

Reviewer #1: Yes

Reviewer #2: Yes

Reviewer #3: Yes

**********

Reviewer #1: Dear Authors,

I am delighted to review your manuscript titled “Study on the Efficiency of Industry–University–Research Collaborative Innovation in China Based on an Improved Two-Stage Game Cross-Efficiency Model Incorporating Shapley Value.” The study develops an enhanced two-stage DEA-based evaluation framework that integrates game cross-efficiency with Shapley value allocation to assess the efficiency of China’s industry–university–research collaborative innovation system across the R&D and technology transfer stages. Using provincial panel data from 2015–2022, the manuscript provides empirical evidence of a persistent structural imbalance characterized by high R&D efficiency but comparatively low technology transfer efficiency, alongside pronounced regional heterogeneity.

My comments go thus:

1. The Introduction is generally well written and clearly motivates the study, but it relies too heavily on single citations to support several authoritative statements. In most cases, key conceptual and contextual claims should be supported by more than one relevant reference to demonstrate adequate engagement with the literature and to strengthen the credibility of the arguments. While multiple citations are not required for every statement, foundational claims would benefit from broader citation support.

2. There are formatting inconsistencies throughout the manuscript that require careful correction. In particular, inconsistent spacing appears after full stops, as observed in the first paragraph of Section 2.1. In addition, many citations lack a space between the final word and the opening square bracket, and citation lists within brackets do not consistently include spaces after commas, for example “[10,11]” instead of “[10, 11]”. These issues should be revised consistently across the entire manuscript to meet standard academic formatting conventions.

3. The first paragraph in Section 2.2 is excessively long and should be divided to improve readability and clarity. More generally, the authors are advised to ensure that no paragraph exceeds approximately 12 lines throughout the manuscript, as overly long paragraphs hinder comprehension and weaken the presentation.

4. Two-stage and network DEA models have been extensively applied across a wide range of domains, particularly in banking, supply chain management, and other service and production systems. The literature review would benefit from a paparagraph or more dedicated to discussion of these broader applications and how such models have been used to capture internal structures and inter-stage relationships before narrowing the focus to industry–university–research collaborative innovation. This would help situate the proposed approach within the wider DEA application landscape and clarify its relevance and transferability to the present study.

5. In line 108, the reference to Sexton et al. is not accompanied by a corresponding bracketed citation. Please add the appropriate square-bracket citation to ensure the source can be clearly and consistently referenced.

6. A similar issue occurs in line 112, where Liang is mentioned without a corresponding bracketed citation. In addition, the same author is inconsistently referred to as “Liang” and “Liang Liang” across lines 110 and 112. These inconsistencies indicate insufficient attention to reference accuracy and citation consistency, and they should be carefully corrected throughout the manuscript.

7. The manuscript focuses on cross-efficiency as a means of distinguishing among efficient DMUs but does not acknowledge or briefly discuss alternative approaches commonly used for this purpose, such as super-efficiency, inverse DEA, or others. While it is acceptable to concentrate on a single method, the authors should at least provide a concise discussion of other established ranking techniques and clarify which improvement they are making.

8. In lines 121–123, the manuscript refers to “subjectivity inherent in traditional weighting methods,” but this claim is not adequately explained or justified. The authors should clarify in what sense these weighting approaches are subjective, for example by explaining whether subjectivity arises from analyst-imposed weights, arbitrary assumptions, or unrestricted weight flexibility in DEA. Without such clarification, the statement remains vague and should either be properly substantiated or revised.

9. Figure 1 is visually weak and does not meet the presentation quality expected for a journal article. The figure should be enlarged, with increased font sizes to ensure readability. If possible, the authors are encouraged to use color and clearer visual differentiation between components to enhance clarity and overall visual appeal.

10. The proposed framework clearly follows a network DEA structure, with multiple stages connected through intermediate outputs, yet the manuscript does not explicitly use the term “network DEA” to characterize the model. The authors should clarify this choice and explain whether the omission is intentional. If the model indeed aligns with the network DEA paradigm, this should be stated explicitly to avoid conceptual ambiguity and to position the contribution more accurately within the DEA literature.

11. Between lines 157–184, the description of the proposed model is insufficiently explicit and should be expanded to better explain the model structure, underlying assumptions, and feasibility. In addition, typographical issues are present, particularly in line 181, where unusual symbols appear between terms instead of standard commas. These errors should be corrected. The authors should also provide clear and complete definitions of all variables and parameters used, and explicitly explain how the equations are logically connected to improve clarity and reproducibility. In addition, some equation numbers are missing. Please revise all, especially how the network is connected and its feasibility.

12. For many of the equations, including Equations (5), (6), (7), and others, the definitions of terms are incomplete or unclear. All symbols, indices, and exponents must be explicitly defined. For instance, in Equation (7), the expression includes s, yet only is defined, with no explanation provided for the exponent . Such omissions undermine the clarity and rigor of the mathematical formulation and should be corrected consistently throughout the manuscript.

13. The manuscript engages a broad DEA methodological space, yet the reference base appears relatively limited for that scope (approximately 24 citations). The literature review and methodological positioning would be strengthened by incorporating more recent and authoritative DEA sources, including key works on network/two-stage DEA, shared-input allocation, cross-efficiency, and game cross-efficiency, as well as recent applications in innovation efficiency contexts. I encourage the authors to expand the bibliography with high-quality journal articles and foundational methodological papers to better demonstrate coverage of the state of the art and to justify the selected modelling choices. You may consider reading and citing the DEA related papers below as they relate to the subject matter.

Inverse DEA for Portfolio Volatility Targeting: Industry Evidence from Taiwan Stock Exchange. International Journal of Financial Studies, 13(4), 192. https://doi.org/10.3390/ijfs13040192

An Integrated Approach to Stock Selection and Ranking: Combining Shannon Entropy Technique, DEA, and Inverse DEA [Article]. Ieee Access, 13, 138851-138866. https://doi.org/10.1109/ACCESS.2025.3586417

Explainable DEA–ensemble approach with golden jackal optimization: efficiency evaluation and prediction for United States information technology firms. Machine Learning with Applications, 23, 100798. https://doi.org/https://doi.org/10.1016/j.mlwa.2025.100798

Reviewer #2: Dear authors,

The manuscript is very interesting, the topic is relevant and the innovation of the manuscript comes from the uses of Shapley values on Two-Stage GCE model.

In terms of methodological innovation, the manuscript incorporates the concept of the Shapley value in IURCI and constructs an improved GCE model integrated with this Shapley value. In methodological terms the manuscript explains in detail all the steps. Indeed, the results are accurate.

The main recommendation is the discussion of the results that are missing and the authors should examine the results and compare them with the other literature results.

Please note along the text in various pages the authors forgot to insert the (year) after the cited reference. Please kindly see: Page 11 line 89 Li Yongjun et al. Please insert (year)

The same after Bingzhou.... Charnes et al…..Sexton et al……on the next pages and all over along the manuscript

Best wishes!

Reviewer #3: The above article proposes a evaluates the efficiency of China’s industry–university–research collaborative innovation (IURCI) system using an improved two-stage game cross-efficiency model integrated with Shapley values. The paper is well-written, although some careful details must be taken into account to be published in Plos One.

Major details

Introduction

Some references are missing that would benefit the paper:

Yongjun Li, Liang Liang,, A Shapley value index on the importance of variables in DEA models, Expert Systems with Applications, Volume 37, Issue 9, 2010, Pages 6287-6292, ISSN 0957-4174, https://doi.org/10.1016/j.eswa.2010.02.093.

Qingxian An, Yao Wen, Tao Ding, Yongli Li, Resource sharing and payoff allocation in a three-stage system: Integrating network DEA with the Shapley value method, Omega,

Volume 85, 2019, Pages 16-25, ISSN 0305-0483,

https://doi.org/10.1016/j.omega.2018.05.008.

Fanyong Meng, Li Wu, Junfei Chu, Allocating the fixed cost based on data envelopment analysis in view of the Shapley value, Expert systems, Volume37, Issue5 Special Issue:Advances in visual analytics and mining visual data, 2020, e12539, https://doi.org/10.1111/exsy.12539

Abing, S.L.N., Barton, M.G.L., Dumdum, M.G.M. et al. Shapley value-based multi-objective data envelopment analysis application for assessing academic efficiency of university departments. J Ind Eng Int 14, 733–746 (2018). https://doi.org/10.1007/s40092-018-0258-6

The introduction should also be extended, considering more recent studies in several DEA stage models. The “black box” mentioned in the text has been widely improved in multiple stage DEA models.

Section 2.2 Where is exactly the novelty with respect to other contributions in the literature stated at “Building on these insights, the present study introduces two major innovations. Theoretically, it incorporates the concept of the Shapley value from cooperative game theory into innovation efficiency evaluation. ”

3.3 Improved Two-Stage GCE Model Incorporating Shapley Value: is the convergence of the model guaranteed?

Results As stated previously, comparison with previous two stage models must be done

Minor details

Please, check for minor typos. Even the title has some spelling mistakes.

**********

what does this mean?). If published, this will include your full peer review and any attached files.

If you choose “no”, your identity will remain anonymous but your review may still be made public.

**Do you want your identity to be public for this peer review?**  For information about this choice, including consent withdrawal, please see our Privacy Policy

Reviewer #1: No

Reviewer #2: No

Reviewer #3: No

**********

[NOTE: If reviewer comments were submitted as an attachment file, they will be attached to this email and accessible via the submission site. Please log into your account, locate the manuscript record, and check for the action link "View Attachments". If this link does not appear, there are no attachment files.]

To ensure your figures meet our technical requirements, please review our figure guidelines: https://journals.plos.org/plosone/s/figures

You may also use PLOS’s free figure tool, NAAS, to help you prepare publication quality figures: https://journals.plos.org/plosone/s/figures#loc-tools-for-figure-preparation.

NAAS will assess whether your figures meet our technical requirements by comparing each figure against our figure specifications.

Attachment Submitted filename: Study on the Efficiency of Industry.docx

# Author comment: Author response to Decision Letter 1

**DOI:** 10.1371/journal.pone.0354999.r002

18 Mar 2026

Respone to Reviewers

I would like to express my sincere gratitude to the reviewers for their feedback on this manuscript. Below, I will address the comments from the three reviewers one by one.

Reviewer #1�

1. The Introduction is generally well written and clearly motivates the study, but it relies too heavily on single citations to support several authoritative statements. In most cases, key conceptual and contextual claims should be supported by more than one relevant reference to demonstrate adequate engagement with the literature and to strengthen the credibility of the arguments. While multiple citations are not required for every statement, foundational claims would benefit from broader citation support.

Response: We appreciate the reviewer’s comments. In response to your feedback, we have reorganized the introduction section. While retaining the original research motivation, core logical framework, and research focus, we have added five high-quality relevant references (specifically, references [1, 2, 4, 6, 7] in this paper) to the existing literature [3, 5, 8]. These additions are primarily intended to further support the foundational, conceptual, and contextual arguments presented in the paper. The supplementary evidence provided by these new references has further enriched the breadth and depth of our literature review, fully demonstrating our in-depth examination of existing research findings. This has effectively enhanced the credibility and strength of our arguments, thereby effectively addressing the previous limitation of the introduction’s narrow range of citations.

2. There are formatting inconsistencies throughout the manuscript that require careful correction. In particular, inconsistent spacing appears after full stops, as observed in the first paragraph of Section 2.1. In addition, many citations lack a space between the final word and the opening square bracket, and citation lists within brackets do not consistently include spaces after commas, for example “[10,11]” instead of “[10, 11]”. These issues should be revised consistently across the entire manuscript to meet standard academic formatting conventions.

Response: To address the formatting issues in this section, we have conducted a thorough, section-by-section and sentence-by-sentence review of the entire manuscript and made consistent revisions: First, we focused on correcting inconsistencies in punctuation and spacing throughout the text to ensure uniformity in punctuation usage and spacing; Second, we conducted a comprehensive review of all citations, adding missing spaces between the final word of a quotation and the opening square bracket. We also standardized the formatting of the citation list within square brackets and corrected issues with the reference citation format to ensure compliance with standard academic norms; Third, we simultaneously reviewed the entire text for any other potential inconsistencies in formatting, implementing comprehensive corrections to effectively enhance the document’s formal rigor.

3. The first paragraph in Section 2.2 is excessively long and should be divided to improve readability and clarity. More generally, the authors are advised to ensure that no paragraph exceeds approximately 12 lines throughout the manuscript, as overly long paragraphs hinder comprehension and weaken the presentation.

Response: In response to your feedback, we have optimized and refined the content of Section 2.2: First, we have reasonably restructured the originally lengthy first paragraph of Section 2.2 to ensure that the paragraph length aligns with your recommendations, significantly improving the readability and clarity of the content; Second, building on the paragraph optimization, we have supplemented the research methods and developmental context of the hyper-efficiency model and the inverse DEA model, further enriching the theoretical foundation of the research methods section and making the argumentation more systematic and in-depth (see lines 136-147 of the manuscript); Third, in the final paragraph of Section 2.2, we have reorganized and refined the innovative aspects of this study, clearly highlighting its core contributions to enable reviewers and readers to quickly grasp the key research highlights (see lines 157-168 of the manuscript).

4. Two-stage and network DEA models have been extensively applied across a wide range of domains, particularly in banking, supply chain management, and other service and production systems. The literature review would benefit from a paparagraph or more dedicated to discussion of these broader applications and how such models have been used to capture internal structures and inter-stage relationships before narrowing the focus to industry–university–research collaborative innovation. This would help situate the proposed approach within the wider DEA application landscape and clarify its relevance and transferability to the present study.

Response: In response to your suggestion, we have added a second paragraph to Section 2.1 (see lines 77-94 of the manuscript) specifically to elaborate on the widespread applications and core roles of two-stage and network DEA models: We clearly articulate the core value of these two models in overcoming the “black box” limitations of traditional DEA and capturing the internal structure of the system as well as the interrelationships among various stages. We discuss their specific applications in the banking sector (measuring the efficiency of various operational stages and aiding operational optimization), supply chain management (characterizing coordination mechanisms among supply chain nodes and optimizing resource allocation), and other service and production systems (such as healthcare and manufacturing, where they identify efficiency bottlenecks). We have cited seven relevant references [17-23] to support these points; At the same time, the text naturally transitions to the field of industry-academia-research collaborative innovation, explaining how the core strengths of these models align with the two-stage R&D process of such collaboration (technology development and technology transfer). It clarifies that the two-stage efficiency measurement framework constructed in this paper represents a concrete application of these models in this field.

5. In line 108, the reference to Sexton et al. is not accompanied by a corresponding bracketed citation. Please add the appropriate square-bracket citation to ensure the source can be clearly and consistently referenced.

Response: The correction has been made on line 134 of the manuscript.

6. A similar issue occurs in line 112, where Liang is mentioned without a corresponding bracketed citation. In addition, the same author is inconsistently referred to as “Liang” and “Liang Liang” across lines 110 and 112. These inconsistencies indicate insufficient attention to reference accuracy and citation consistency, and they should be carefully corrected throughout the manuscript.

Response: We have implemented targeted corrections regarding this issue: First, we have standardized the citation format for the two relevant papers by Professor Liang Liang, supplementing and refining them in strict accordance with academic citation standards. The specific revisions can be found on lines 148 and 150 of the manuscript; We have standardized the author’s name, correcting the inconsistent references to “Liang” and “Liang Liang” throughout the text to “Liang Liang” to ensure consistency in author attribution; second, taking this as an opportunity, we have conducted a comprehensive review and standardized revision of the citations and author attribution throughout the entire manuscript, focusing on issues such as missing citations, inconsistent author names, and non-compliant citation formats, to prevent such problems from recurring.

7. The manuscript focuses on cross-efficiency as a means of distinguishing among efficient DMUs but does not acknowledge or briefly discuss alternative approaches commonly used for this purpose, such as super-efficiency, inverse DEA, or others. While it is acceptable to concentrate on a single method, the authors should at least provide a concise discussion of other established ranking techniques and clarify which improvement they are making.

Response: In response to your suggestion, we have added relevant content to the second paragraph of Section 2.2 (lines 136-147), specifically to discuss alternative methods for ranking efficient DMUs and to justify the rationale behind the method selected in this paper: We clearly introduce two commonly used methods for efficient DMU ranking—the super-efficiency DEA model and the inverse DEA model—and explain the core principles, advantages, and limitations of each: the super-efficiency model is simple to compute and highly interpretable, but struggles to account for the interdependent effects across different stages of a multi-stage system; the inverse DEA model provides specific optimization directions but is highly sensitive to data and prone to being influenced by outliers; At the same time, the paper clearly explains the rationale for selecting the cross-efficiency model, emphasizing its advantage of incorporating a mutual evaluation mechanism among DMUs. This approach not only aligns with the interactive relationships among various entities in industry-academia-research collaborative innovation but also facilitates integration with game theory. It addresses the shortcomings of the super-efficiency and inverse DEA models in depicting entity interactions, thereby clearly highlighting the rationality of the methodological choice and the value of the proposed improvements, making the justification of the research methodology more comprehensive and persuasive.

8. In lines 121–123, the manuscript refers to “subjectivity inherent in traditional weighting methods,” but this claim is not adequately explained or justified. The authors should clarify in what sense these weighting approaches are subjective, for example by explaining whether subjectivity arises from analyst-imposed weights, arbitrary assumptions, or unrestricted weight flexibility in DEA. Without such clarification, the statement remains vague and should either be properly substantiated or revised.

Response: Since the “subjectivity inherent in traditional weighting methods” mentioned in the manuscript is not the primary distinction between the new model and traditional models, we have revised the analysis in the literature review section to highlight key points and strike an appropriate balance between detail and brevity, thereby clarifying the description of the paper’s contributions and innovations.

9. Figure 1 is visually weak and does not meet the presentation quality expected for a journal article. The figure should be enlarged, with increased font sizes to ensure readability. If possible, the authors are encouraged to use color and clearer visual differentiation between components to enhance clarity and overall visual appeal.

Response: In response to your suggestion, we have revised Figure 1. The revised version can be found on line 196 of the manuscript: First, we have enlarged the image to ensure greater overall clarity. Second, we have increased the font size within the image to address the issue of poor readability, making it easier for reviewers and readers to clearly view the content. Third, in accordance with the journal’s requirements, we have optimized the visual presentation of the figure, enhanced the visual distinction between its various components, and further improved its clarity and overall appearance to ensure it meets the image requirements of《PLOS ONE》.

10. The proposed framework clearly follows a network DEA structure, with multiple stages connected through intermediate outputs, yet the manuscript does not explicitly use the term “network DEA” to characterize the model. The authors should clarify this choice and explain whether the omission is intentional. If the model indeed aligns with the network DEA paradigm, this should be stated explicitly to avoid conceptual ambiguity and to position the contribution more accurately within the DEA literature.

Response: It should be clarified that the two-stage efficiency evaluation framework constructed in this paper is essentially an extension of the network DEA model. By decomposing the collaborative innovation process among industry, academia, and research institutions into two sequential stages—technology R&D and technology transfer—it accurately captures the internal flow mechanisms of the innovation process. The previous lack of explicit use of the term “network DEA” may have led to ambiguity regarding the model’s theoretical positioning. In this revised version, we clarify its connection to network DEA, further highlighting the research method’s positioning and value within the DEA literature.

11. Between lines 157–184, the description of the proposed model is insufficiently explicit and should be expanded to better explain the model structure, underlying assumptions, and feasibility. In addition, typographical issues are present, particularly in line 181, where unusual symbols appear between terms instead of standard commas. These errors should be corrected. The authors should also provide clear and complete definitions of all variables and parameters used, and explicitly explain how the equations are logically connected to improve clarity and reproducibility. In addition, some equation numbers are missing. Please revise all, especially how the network is connected and its feasibility.

Response: In response to the issues you raised, we have completed a systematic review and revision: First, we have thoroughly reviewed and optimized this section, removing redundant content, streamlining the wording, and expanding the model description; second, we have corrected formatting issues by replacing non-standard symbols used between terms with standard symbols, and simultaneously identified and corrected other potential formatting inconsistencies in this paragraph; Third, we have re-edited the model content, revising and refining all variables and parameters used in the model, and clearly explaining the logical relationships between the equations to make the model’s derivation more coherent and its structure clearer; Fourth, we have added missing equation numbers to ensure consistent and standardized numbering across all equations, further enhancing the rigor of the manuscript.

12. For many of the equations, including Equations (5), (6), (7), and others, the definitions of terms are incomplete or unclear. All symbols, indices, and exponents must be explicitly defined. For instance, in Equation (7), the expression includes s, yet only is defined, with no explanation provided for the exponent . Such omissions undermine the clarity and rigor of the mathematical formulation and should be corrected consistently throughout the manuscript.

Response: In response to your comments, we have completed a systematic revision of the research model section in Chapter 3: We have focused on reorganizing and optimizing the model and related content in Sections 3.1, 3.2, 3.3 (see lines 170–274 of the manuscript), while also conducting a comprehensive review of all term definitions and variable symbols throughout the manuscript. We have supplemented and refined each one to ensure that all symbols, indices, and exponents in the equations are clearly and explicitly explained, thereby thoroughly correcting omissions such as the unexplained exponent in Equation (7). Furthermore, we have conducted a comprehensive review of the mathematical expressions throughout the manuscript to ensure consistency and standardization in the definitions of terms and explanations of variable symbols. This has effectively enhanced the clarity and academic rigor of the mathematical presentation and prevents such issues from recurring.

13. The manuscript engages a broad DEA methodological space, yet the reference base appears relatively limited for that scope (approximately 24 citations). The literature review and methodological positioning would be strengthened by incorporating more recent and authoritative DEA sources, including key works on network/two-stage DEA, sh

Attachment Submitted filename: Response to Reviewers.docx

# Peer review: Decision Letter 1

**DOI:** 10.1371/journal.pone.0354999.r003

**Authors:** Hongbum Kim

20 Apr 2026

Dear Dr. Ji,

Thank you for submitting your manuscript to PLOS ONE. After careful consideration, we feel that it has merit but does not fully meet PLOS ONE’s publication criteria as it currently stands. Therefore, we invite you to submit a revised version of the manuscript that addresses the points raised during the review process.

Please submit your revised manuscript by Jun 04 2026 11:59PM. If you will need more time than this to complete your revisions, please reply to this message or contact the journal office at plosone@plos.org. When you're ready to submit your revision, log on to https://www.editorialmanager.com/pone/ and select the 'Submissions Needing Revision' folder to locate your manuscript file.

- A letter that responds to each point raised by the academic editor and reviewer(s). You should upload this letter as a separate file labeled 'Response to Reviewers'.
- A marked-up copy of your manuscript that highlights changes made to the original version. You should upload this as a separate file labeled 'Revised Manuscript with Track Changes'.
- An unmarked version of your revised paper without tracked changes. You should upload this as a separate file labeled 'Manuscript'.

If you would like to make changes to your financial disclosure, please include your updated statement in your cover letter. Guidelines for resubmitting your figure files are available below the reviewer comments at the end of this letter.

If applicable, we recommend that you deposit your laboratory protocols in protocols.io to enhance the reproducibility of your results. Protocols.io assigns your protocol its own identifier (DOI) so that it can be cited independently in the future. For instructions see: https://journals.plos.org/plosone/s/submission-guidelines#loc-laboratory-protocols. Additionally, PLOS ONE offers an option for publishing peer-reviewed Lab Protocol articles, which describe protocols hosted on protocols.io. Read more information on sharing protocols at https://plos.org/protocols?utm_medium=editorial-email&utm_source=authorletters&utm_campaign=protocols.

As the corresponding author, your ORCID iD is verified in the submission system and will appear in the published article. PLOS supports the use of ORCID, and we encourage all coauthors to register for an ORCID iD and use it as well. Please encourage your coauthors to verify their ORCID iD within the submission system before final acceptance, as unverified ORCID iDs will not appear in the published article. *Only*  the individual author can complete the verification step; PLOS staff *cannot*  verify ORCID iDs on behalf of authors.

We look forward to receiving your revised manuscript.

Kind regards,

Hongbum Kim, Ph.D.

Academic Editor

PLOS One

Journal Requirements:

If the reviewer comments include a recommendation to cite specific previously published works, please review and evaluate these publications to determine whether they are relevant and should be cited. There is no requirement to cite these works unless the editor has indicated otherwise.

Additional Editor Comments (if provided):

Despite the positive evaluation of the revised manuscript by Reviewers, it appears that several issues have not yet been adequately addressed.

#2-1. Do you really believe that a sufficient discussion and comparison was done with just a single additional reference? In result analysis section, the references supporting the authors’ arguments is generally insufficient. Conduct the discussion and comparison by including a variety of existing studies. This applies to entire Section 4.

#3-5. Do you really believe that you have corrected the typo in the title?

Additionally, the following points should be addressed.

1. Avoid excessive use of quotation marks. It gives the impression that the manuscript is a product of GenAI. I do not believe that any of the currently used quotation marks are necessary.

2. Although this journal is at the SCIE level, most of the references cited are either not SCIE-level or are limited to domestic journals in China. Except for the critical research, please replace most of the references with SCIE-level ones. As a current manuscript, concerns about the reliability and quality of the references are raised.

3. Although this research is regarding IURCI, there is no literature review on innovation theory to support the research. Please add innovation studies literature as a subsection of Literature, and also use them in the result analysis and discussion parts.

4. I do not believe that the name of Sections 2.1 and 2.2 are appropriate. Please revise them to accurately represent the main text.

5. As there are many mathematical formulas, please review once more to ensure that formulas including superscripts and subscripts have been written correctly.

6. In Table 2, the variability of the weighting coefficients across years is quite large. In order to ensure the reliability of these results, please consider additional tests.

7. Indicate the number of game iterations.

[Note: HTML markup is below. Please do not edit.]

Reviewers' comments:

Reviewer's Responses to Questions

**Comments to the Author**

Reviewer #1: All comments have been addressed

Reviewer #2: All comments have been addressed

Reviewer #3: All comments have been addressed

**********

2. Is the manuscript technically sound, and do the data support the conclusions?

Reviewer #1: Yes

Reviewer #2: Yes

Reviewer #3: Yes

**********

3. Has the statistical analysis been performed appropriately and rigorously?

Reviewer #1: Yes

Reviewer #2: Yes

Reviewer #3: Yes

**********

4. Have the authors made all data underlying the findings in their manuscript fully available?

The PLOS Data policy

Reviewer #1: Yes

Reviewer #2: Yes

Reviewer #3: Yes

**********

5. Is the manuscript presented in an intelligible fashion and written in standard English?

Reviewer #1: Yes

Reviewer #2: Yes

Reviewer #3: Yes

**********

Reviewer #1: The revised copy has really enhanced the quality of the manuscript and it is now considered okay for publication

Reviewer #2: Dear Authors,

The authors carry out all the recomendations and the paper should be approved.

Wishing you the best wishes with the referred paper

Reviewer #3: Congratulations to the authors for their effort in improving the paper. I found now suitable to be published in Plos One

**********

what does this mean?). If published, this will include your full peer review and any attached files.

If you choose “no”, your identity will remain anonymous but your review may still be made public.

**Do you want your identity to be public for this peer review?**  For information about this choice, including consent withdrawal, please see our Privacy Policy

Reviewer #1: No

Reviewer #2: No

Reviewer #3: No

**********

[NOTE: If reviewer comments were submitted as an attachment file, they will be attached to this email and accessible via the submission site. Please log into your account, locate the manuscript record, and check for the action link "View Attachments". If this link does not appear, there are no attachment files.]

To ensure your figures meet our technical requirements, please review our figure guidelines: https://journals.plos.org/plosone/s/figures

You may also use PLOS’s free figure tool, NAAS, to help you prepare publication quality figures: https://journals.plos.org/plosone/s/figures#loc-tools-for-figure-preparation.

NAAS will assess whether your figures meet our technical requirements by comparing each figure against our figure specifications.

# Author comment: Author response to Decision Letter 2

**DOI:** 10.1371/journal.pone.0354999.r004

7 Jun 2026

1. Do you really believe that a sufficient discussion and comparison was done with just a single additional reference? In result analysis section, the references supporting the authors’ arguments is generally insufficient. Conduct the discussion and comparison by including a variety of existing studies. This applies to entire Section 4.

Response�We sincerely accept your comments and have revised and expanded Chapter 4 accordingly. The revision no longer relies on a limited number of references for discussion; instead, we have incorporated a broad range of existing relevant studies and conducted comprehensive comparative analysis and in-depth discussion.

In the revised chapter, we systematically explain the imbalance in two-stage resource allocation and the heterogeneous characteristics of regional efficiency by integrating innovation value chain theory, triple helix theory, open innovation theory, and absorptive capacity theory. Meanwhile, we introduce cutting-edge research findings on knowledge barriers, knowledge flow, and regional innovation development, and conduct horizontal comparisons with the empirical results of this study. We have supplemented sufficient literature support for the research conclusions and improved the comparative discussions with existing studies throughout Chapter 2, further enhancing the academic rigor and persuasiveness of the result analysis.

2. Do you really believe that you have corrected the typo in the title?

Response�Yes, we have carefully corrected the spelling error in the title. In addition, we have conducted a comprehensive spelling check and proofreading throughout the entire manuscript to ensure that no similar errors appear.

3.Avoid excessive use of quotation marks. It gives the impression that the manuscript is a product of GenAI. I do not believe that any of the currently used quotation marks are necessary.

Response�We fully agree with this comment. We have removed all unnecessary quotation marks throughout the manuscript and standardized the usage of all punctuation marks in strict accordance with academic writing conventions.

4.Although this journal is at the SCIE level, most of the references cited are either not SCIE-level or are limited to domestic journals in China. Except for the critical research, please replace most of the references with SCIE-level ones. As a current manuscript, concerns about the reliability and quality of the references are raised.

Response�Thank you very much for your valuable comments. We have comprehensively optimized the reference list, replaced and removed some domestic Chinese journal articles, and supplemented high-quality SCIE-level literature. The revised 42 references include 12 Chinese articles and 30 English articles, which we hope will enhance the academic quality and reliability of the citations.

5.Although this research is regarding IURCI, there is no literature review on innovation theory to support the research. Please add innovation studies literature as a subsection of Literature, and also use them in the result analysis and discussion parts.

Response�Thank you very much for your pertinent suggestions. We have added a new Subsection 2.1 titled “Innovation theory perspectives on IURCI ”in the literature review chapter. This subsection systematically supplements three core theories that are highly consistent with IURCI: innovation value chain theory, open innovation and absorptive capacity theory, and triple helix theory. Meanwhile, the relevant theoretical content has been reasonably integrated into the results and discussion section, so as to consolidate the theoretical foundation of this study.

6.I do not believe that the name of Sections 2.1 and 2.2 are appropriate. Please revise them to accurately represent the main text.

Response�We have carefully adopted this revision suggestion. The title of the original Section 2.1 (now renumbered as Section 2.2) has been revised to Indicator systems and Shapley value applictions in IURCI efficiency evaluation. The original Section 2.2 (now renumbered as Section 2.3) has also been revised and renamed as“ Efficiency evaluation methods for IURCI”.

5.As there are many mathematical formulas, please review once more to ensure that formulas including superscripts and subscripts have been written correctly.

Response�We have thoroughly checked and organized all mathematical formulas throughout the manuscript. The subscripts, superscripts, and various symbol formats have been verified one by one to ensure that all formulas are written in a standardized and accurately expressed manner.

6.In Table 2, the variability of the weighting coefficients across years is quite large. In order to ensure the reliability of these results, please consider additional tests.

Response�Thank you very much for your valuable comments. The large differences in the Shapley value weights across years in Table 2 are objective results calculated from the actual data. We have rechecked and recalculated the raw data and confirmed that these differences are not caused by measurement errors. In addition, we have explained the underlying reasons for such differences in the manuscript. The fluctuations in the weights stem from real changes in various innovation input and output indicators. The substantial differences in certain years are mainly due to drastic short-term changes in some indicators driven by policy adjustments, which led to sharp increases in their corresponding weights. For example, as mentioned in Session 4.1: “In contrast, the contribution of industrial enterprises above a designated size engaged in R&D activities experienced a sharp increase only in 2019 (weight coefficient 0.2736), likely resulting from the temporary strengthening of enterprises’leading role in innovation under policy incentives”. Through repeated calculations and analyses, we confirm that the weight coefficients presented in Table 2 are reasonable and reliable.

7.Indicate the number of game iterations.

Response�In the empirical calculation process, a clear convergence criterion is established in advance: when the absolute differences of both the overall efficiency and the sub-stage efficiencies between two consecutive iterations are all less than 10-4, all DMUs are considered to have reached a convergent state, and the calculation results are stable and reliable. It should be noted that the actual number of iterations for each DMU is not exactly the same, so a unified number of iterations cannot be provided. Once the above convergence condition is met, the iteration process terminates automatically.

Attachment Submitted filename: Response_to_Reviewers_auresp_2.docx

# Editor report: Decision Letter 2

**DOI:** 10.1371/journal.pone.0354999.r005

**Authors:** Hongbum Kim

1 Jul 2026

Dear Dr. Ji,

Thank you for submitting your manuscript to PLOS ONE. After careful consideration, we feel that it has merit but does not fully meet PLOS ONE’s publication criteria as it currently stands. Therefore, we invite you to submit a revised version of the manuscript that addresses the points raised during the review process.

Please submit your revised manuscript by Aug 15 2026 11:59PM. If you will need more time than this to complete your revisions, please reply to this message or contact the journal office at plosone@plos.org. When you're ready to submit your revision, log on to https://www.editorialmanager.com/pone/ and select the 'Submissions Needing Revision' folder to locate your manuscript file.

- A letter that responds to each point raised by the academic editor and reviewer(s). You should upload this letter as a separate file labeled 'Response to Reviewers'.
- A marked-up copy of your manuscript that highlights changes made to the original version. You should upload this as a separate file labeled 'Revised Manuscript with Track Changes'.
- An unmarked version of your revised paper without tracked changes. You should upload this as a separate file labeled 'Manuscript'.

If you would like to make changes to your financial disclosure, please include your updated statement in your cover letter. Guidelines for resubmitting your figure files are available below the reviewer comments at the end of this letter.

If applicable, we recommend that you deposit your laboratory protocols in protocols.io to enhance the reproducibility of your results. Protocols.io assigns your protocol its own identifier (DOI) so that it can be cited independently in the future. For instructions see: https://journals.plos.org/plosone/s/submission-guidelines#loc-laboratory-protocols. Additionally, PLOS ONE offers an option for publishing peer-reviewed Lab Protocol articles, which describe protocols hosted on protocols.io. Read more information on sharing protocols at https://plos.org/protocols?utm_medium=editorial-email&utm_source=authorletters&utm_campaign=protocols.

As the corresponding author, your ORCID iD is verified in the submission system and will appear in the published article. PLOS supports the use of ORCID, and we encourage all coauthors to register for an ORCID iD and use it as well. Please encourage your coauthors to verify their ORCID iD within the submission system before final acceptance, as unverified ORCID iDs will not appear in the published article. *Only*  the individual author can complete the verification step; PLOS staff *cannot*  verify ORCID iDs on behalf of authors.

We look forward to receiving your revised manuscript.

Kind regards,

Hongbum Kim, Ph.D.

Academic Editor

PLOS One

Journal Requirements:

1. If the reviewer comments include a recommendation to cite specific previously published works, please review and evaluate these publications to determine whether they are relevant and should be cited. There is no requirement to cite these works unless the editor has indicated otherwise.

2. Please review your reference list to ensure that it is complete and correct. If you have cited papers that have been retracted, please include the rationale for doing so in the manuscript text, or remove these references and replace them with relevant current references. Any changes to the reference list should be mentioned in the rebuttal letter that accompanies your revised manuscript. If you need to cite a retracted article, indicate the article’s retracted status in the References list and also include a citation and full reference for the retraction notice.

Additional Editor Comments:

All issues are relevantly addressed, except for literature reviews on innovation. Only limited papers were merely mentioned for each innovation theory, which was not expected when I left comment. While it would be beneficial to enrich the relevant discussions in each subsection, rather I propose merging all sub-sections into section 2.1 only and then literature on innovation should be logically reviewed in a single section. Final acceptance will be on the condition that the aforementioned issue is sufficiently addressed.

[Note: HTML markup is below. Please do not edit.]

[NOTE: If reviewer comments were submitted as an attachment file, they will be attached to this email and accessible via the submission site. Please log into your account, locate the manuscript record, and check for the action link "View Attachments". If this link does not appear, there are no attachment files.]

To ensure your figures meet our technical requirements, please review our figure guidelines: https://journals.plos.org/plosone/s/figures

You may also use PLOS’s free figure tool, NAAS, to help you prepare publication quality figures: https://journals.plos.org/plosone/s/figures#loc-tools-for-figure-preparation.

NAAS will assess whether your figures meet our technical requirements by comparing each figure against our figure specifications.

# Author comment: Author response to Decision Letter 3

**DOI:** 10.1371/journal.pone.0354999.r006

13 Jul 2026

Response to Reviewers

1. If the reviewer comments include a recommendation to cite specific previously published works, please review and evaluate these publications to determine whether they are relevant and should be cited. There is no requirement to cite these works unless the editor has indicated otherwise.

Response�Thank you for your reminder. We have thoroughly combed through the entire manuscript to ensure that only references highly relevant to this research are cited.

2. Please review your reference list to ensure that it is complete and correct. If you have cited papers that have been retracted, please include the rationale for doing so in the manuscript text, or remove these references and replace them with relevant current references. Any changes to the reference list should be mentioned in the rebuttal letter that accompanies your revised manuscript. If you need to cite a retracted article, indicate the article’s retracted status in the References list and also include a citation and full reference for the retraction notice.

Response�Thank you for your valuable comments. We have thoroughly double-checked all references throughout the manuscript and confirmed that no retracted literature has been cited.

3.All issues are relevantly addressed, except for literature reviews on innovation. Only limited papers were merely mentioned for each innovation theory, which was not expected when I left comment. While it would be beneficial to enrich the relevant discussions in each subsection, rather I propose merging all sub-sections into section 2.1 only and then literature on innovation should be logically reviewed in a single section. Final acceptance will be on the condition that the aforementioned issue is sufficiently addressed.

Response�Thank you for your valuable comments. We have reorganized the content of Section 2.1 Innovation theory perspectives on IURCI in the original manuscript. The innovation theories distributed across the three subsections are integrated into a unified section, and additional key supporting literatures are supplemented accordingly. The revised passages can be found on Lines 70-106, Line166-178,Line 319, Line 330, Line 356 and Line 380 of the revised manuscript.

Data Availability

The panel dataset supporting the findings of this study covers indicators of industry-university-research collaborative innovation across 2015-2022, with all raw data sourced from China Statistical Yearbook. The dataset has been publicly deposited in Figshare , with the unique Figshare identifier https://doi.org/10.6084/m9.figshare.32955860.

This dataset contains all indicator variables for IURCI from 2015 to 2022, as well as executable codes for replicating the two-stage DEA game cross-efficiency and Shapley value model calculations.

The ORCID ID information of the authors is listed as follows:

Xiaoling Ji: ORCID ID: 0009-0008-0124-0870;

Xiaoqing Li: ORCID ID: 0009-0008-3095-010X.

Attachment Submitted filename: Response_to_Reviewers_auresp_3.docx

# Editor report: Decision Letter 3

**DOI:** 10.1371/journal.pone.0354999.r007

**Authors:** Hongbum Kim

16 Jul 2026

Study on the Efficiency of Industry-University-Research Collaborative Innovation in China Based on a Two-Stage Game Cross-Efficiency Model Incorporating Shapley Value

PONE-D-25-65565R3

Dear Dr. Ji,

We’re pleased to inform you that your manuscript has been judged scientifically suitable for publication and will be formally accepted for publication once it meets all outstanding technical requirements.

Within one week, you’ll receive an e-mail detailing the required amendments. When these have been addressed, you’ll receive a formal acceptance letter and your manuscript will be scheduled for publication.

An invoice will be generated when your article is formally accepted. Please note, if your institution has a publishing partnership with PLOS and your article meets the relevant criteria, all or part of your publication costs will be covered. Please make sure your user information is up-to-date by logging into Editorial Manager at Editorial Manager® and clicking the ‘Update My Information' link at the top of the page. For questions related to billing, please contact billing support.

If your institution or institutions have a press office, please notify them about your upcoming paper to help maximize its impact. If they’ll be preparing press materials, please inform our press team as soon as possible -- no later than 48 hours after receiving the formal acceptance. Your manuscript will remain under strict press embargo until 2 pm Eastern Time on the date of publication. For more information, please contact onepress@plos.org.

Kind regards,

Hongbum Kim, Ph.D.

Academic Editor

PLOS One

Additional Editor Comments (optional):

Thank you for your efforts on revising the manuscript. All issues are relevantly addressed.

Reviewers' comments:

# Editor report: Acceptance letter

**DOI:** 10.1371/journal.pone.0354999.r008

**Authors:** Hongbum Kim

PONE-D-25-65565R3

PLOS One

Dear Dr. Ji,

I'm pleased to inform you that your manuscript has been deemed suitable for publication in PLOS One. Congratulations! Your manuscript is now being handed over to our production team.

At this stage, our production department will prepare your paper for publication. This includes ensuring the following:

* All references, tables, and figures are properly cited

* All relevant supporting information is included in the manuscript submission,

* There are no issues that prevent the paper from being properly typeset

You will receive further instructions from the production team, including instructions on how to review your proof when it is ready. Please keep in mind that we are working through a large volume of accepted articles, so please give us a few days to review your paper and let you know the next and final steps.

Lastly, if your institution or institutions have a press office, please let them know about your upcoming paper now to help maximize its impact. If they'll be preparing press materials, please inform our press team within the next 48 hours. Your manuscript will remain under strict press embargo until 2 pm Eastern Time on the date of publication. For more information, please contact onepress@plos.org.

You will receive an invoice from PLOS for your publication fee after your manuscript has reached the completed accept phase. If you receive an email requesting payment before acceptance or for any other service, this may be a phishing scheme. Learn how to identify phishing emails and protect your accounts at https://explore.plos.org/phishing.

If we can help with anything else, please email us at customercare@plos.org.

Thank you for submitting your work to PLOS ONE and supporting open access.

Kind regards,

PLOS ONE Editorial Office Staff

on behalf of

Professor Hongbum Kim

Academic Editor

PLOS One
